# Supplementary material for: Outcompeting p53-Mutant Cells in the Normal Esophagus by Redox Manipulation
Source: Cell Stem Cell. 2019 Sep 5;25(3):329–341.e6. doi: 10.1016/j.stem.2019.06.011 (PMC6739485; doi:10.1016/j.stem.2019.06.011)
Supplement: Document S1. Figures S1–S6 and Tables S1 and S2 [file mmc1.pdf]

Cell Stem Cell, Volume *25*

Supplemental Information

Outcompeting *p53*-Mutant Cells in the Normal Esophagus by Redox Manipulation

David Fernandez-Antoran, Gabriel Piedrafita, Kasumi Murai, Swee Hoe Ong, Albert Herms, Christian Frezza, and Philip H. Jones

Supplemental Material for:

Outcompeting p53-mutant cells in normal esophagus by redox manipulation

David Fernandez-Antoran, Gabriel Piedrafita, Kasumi Murai, Swee Hoe Ong, Albert Herms, Christian Frezza, Philip H. Jones

This PDF file includes:

Supplementary Figures and Legends

Figure S1. Related to Figures 2, 5 and 6

Figure S2. Related to Figure 2

Figure S3. Related to Figure 2

Figure S4. Related to Figures 2 and 3

Figure S5. Related to Figure 5

Figure S6. Related to Figure 6

Supplementary Tables and Data

Table S1, related to Figures 3 and 5: Number of quantified mitochondria in Figure 3 and Figure 5

Table S2, related to Figures 2,3,4, 5, 6: Number of quantified clones per condition per time point in Figures 2, 4, 5, 6 and Figure S3.

Supplementary Figures and Legends

Figure S1

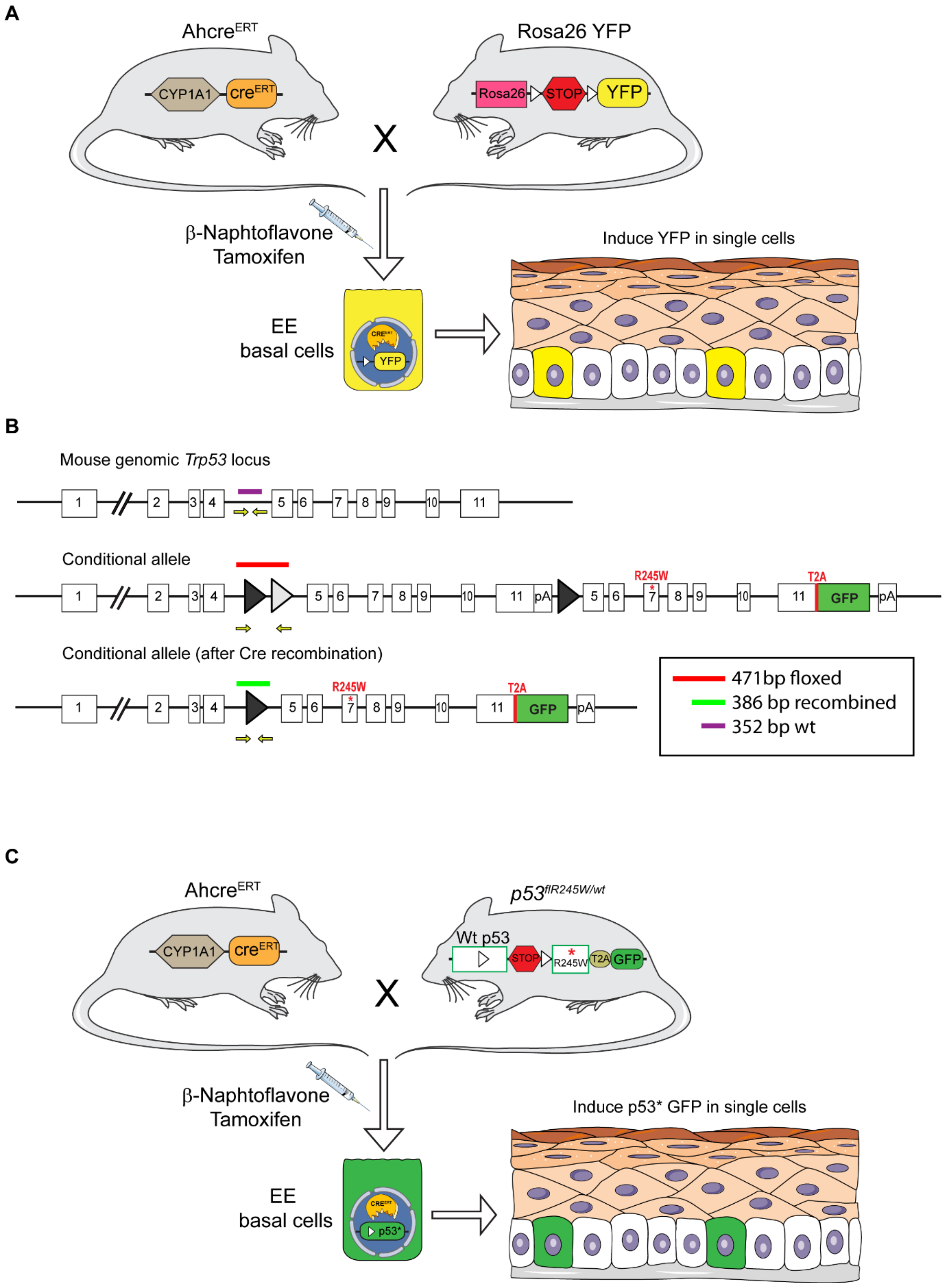

Figure S1. Transgenic mouse models. Related to Figures 2, 5 and 6.

(A) Generation of double transgenic *Ahcre*^{ERT}*R26*$^{flEYFP/wt}$*,* (*RYFP)* mice for lineage cell tracing experiments. *Yfp* (yellow) is targeted to the *Rosa26* locus downstream of a 'stop' cassette (red). Following *cre* induction, the stop cassette is excised and YFP protein expressed in a small proportion of esophageal basal cells. (B) Gene targeting strategy for generation of *p53*$^{R245W-GFP}$ mice, in which a conditional mutant allele of *Trp53*^{R245W} (*p53*)* linked to the reporter protein GFP is targeted to the *Trp53* locus (Murai et al., 2018). (C) Generation of *Ahcre*^{ERT} *p53** mice. Following *cre* induction, *Trp53* wild type exons 5 to 11 are excised and p53* mutant allele and GFP reporter protein are expressed in a small proportion of oesophageal basal cells.

Figure S2

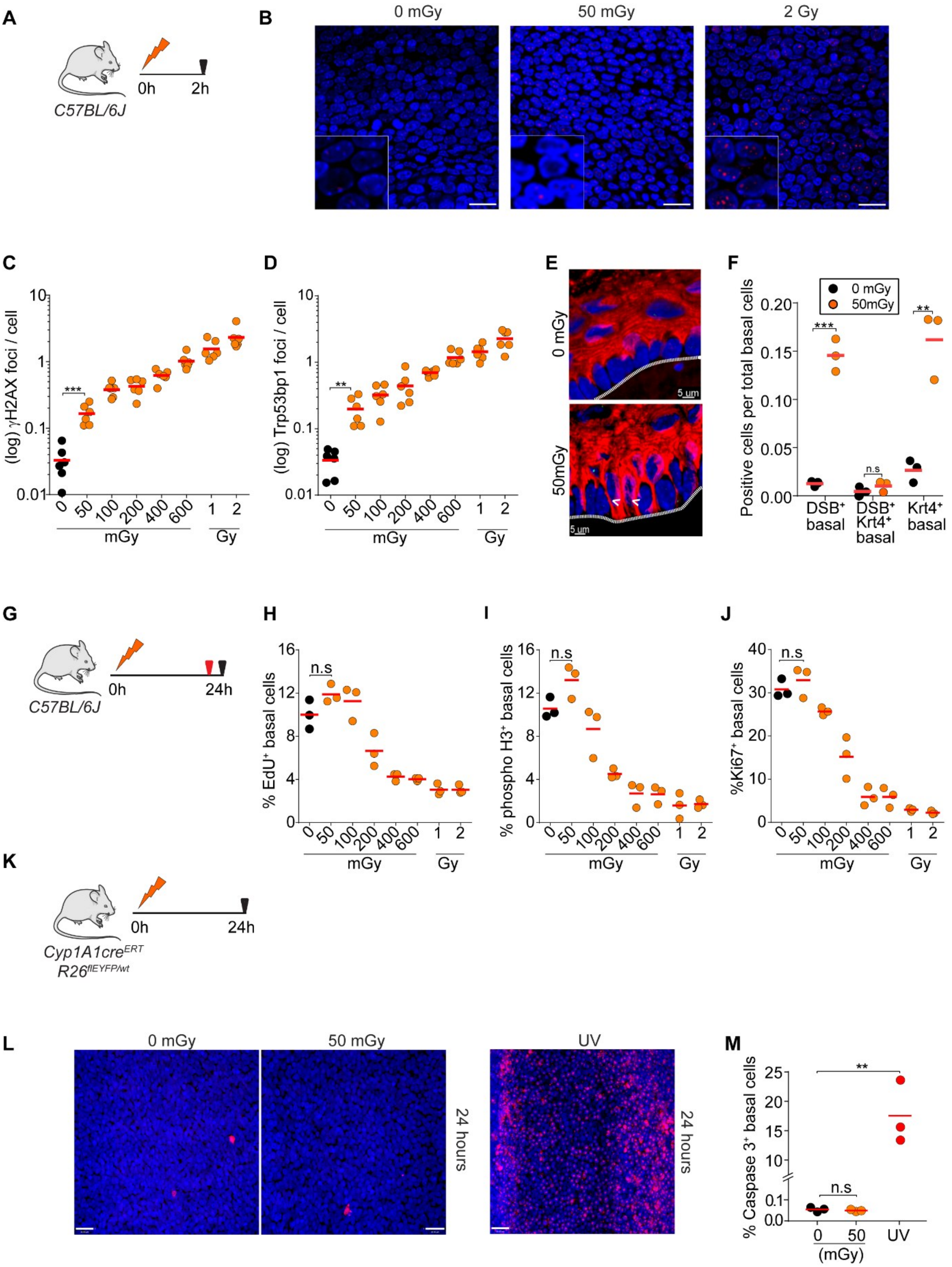
A
0h
2h
C57BL/6J
B
0 mGy
50 mGy
2 Gy
C
(log) γH2AX foci / cell
mGy
Gy
D
(log) Trp53bp1 foci / cell
E
0 mGy
50mGy
F
Positive cells per total basal cells
0 mGy
50mGy
DSB+ basal
DSB+ Krt4+ basal
Krt4+ basal
n.s
G
0h
24h
C57BL/6J
H
% EdU+ basal cells
n.s
I
% phospho H3+ basal cells
J
%Ki67+ basal cells
K
0h
24h
Cyp1A1creERT R26flEYFP/wt
L
0 mGy
50 mGy
UV
24 hours
M
% Caspase 3+ basal cells
(mGy)
UV

Figure S2. Dose response of EE to whole body ionizing radiation. Related to Figure 2.

(A) Protocol to study DNA damage dose response of basal cells in EE. Wild type *C57BL/6J* mice were irradiated and tissue collected 2 hours later. (B) Top down views of confocal z stacks of *Cyp1A1cre*^{ERT} *Rosa26*$^{flEYFP/wt}$ mice EE wholemounts stained for DNA (DAPI, blue) showing nuclear foci γH2AX (double strand DNA break marker, red) 2 hours after 0 mGy, 50 mGy or 2Gy exposures. DAPI (blue). Scale bars, 20 μm. (C and D) Nuclear foci/basal cell of γH2AX (C) and *Trp53bp1* (D) after whole body irradiation with doses shown. At least 250 basal cells per mouse in 6 mice at each dose were analyzed by confocal imaging. ***p<0.001 and **p<0.01 (unpaired t test). (E) OCT embedded 10 μm thickness cryosections of EE of Cyp1A1^{creERT} Rosa26$^{flEYFP/wt}$ 2 hours after 0 mGy or 50 mGy LDIR. Krt4 (red), Trp53bp1 (DSB, green) and DAPI (blue). Dashed lines indicate basement membrane. Arrowheads show Krt4 positive basal cells. Scale bars, 5μm. (F) Quantification of DSB^{+}, DSB^{+} Krt4^{+} and Krt4^{+} basal cells shown in (E). Points are mean values from individual mice. ***p<0.001, **p<0.01, n.s not significant, (unpaired t test), n=3 mice per condition. (G) Protocol to study the effect of ionizing radiation on cell proliferation. Wild-type *C57BL/6J* mice were irradiated as in (A) and culled 24 hours later. One hour before culling, animals were injected with EdU intraperitoneally. (H-J) Percentage of basal cells positive for the proliferation markers, EdU (H) phospho-Histone 3 (I) and Ki67 (J), 24 hours after exposure to different doses of ionizing radiation. 3 mice per condition were analyzed by confocal imaging. n.s not significant (unpaired t test). (K-M) Apoptosis *in vivo* assay after LDIR. (K) Experimental protocol. *Cyp1A1cre*^{ERT}*Rosa26*$^{flEYFP/wt}$ mice were irradiated with 0 or 50 mGy LDIR and EE stained 24 hours after LDIR exposure. As a positive control tissue was exposed to ultraviolet radiation and maintained in explant culture for 24 hours prior to staining (Clayton et al., 2007). (L) Confocal images of basal layer of EE stained for activated Caspase 3 (red) and DAPI (blue). Scale bars, 30 μm. (M) Quantification of the percentage of Caspase 3^{+} cells shown in (J). At least 3000 basal cells in 3 mice were analyzed per condition by confocal imaging. ** p<0.01, n.s. not significant (unpaired t test).

Figure S3

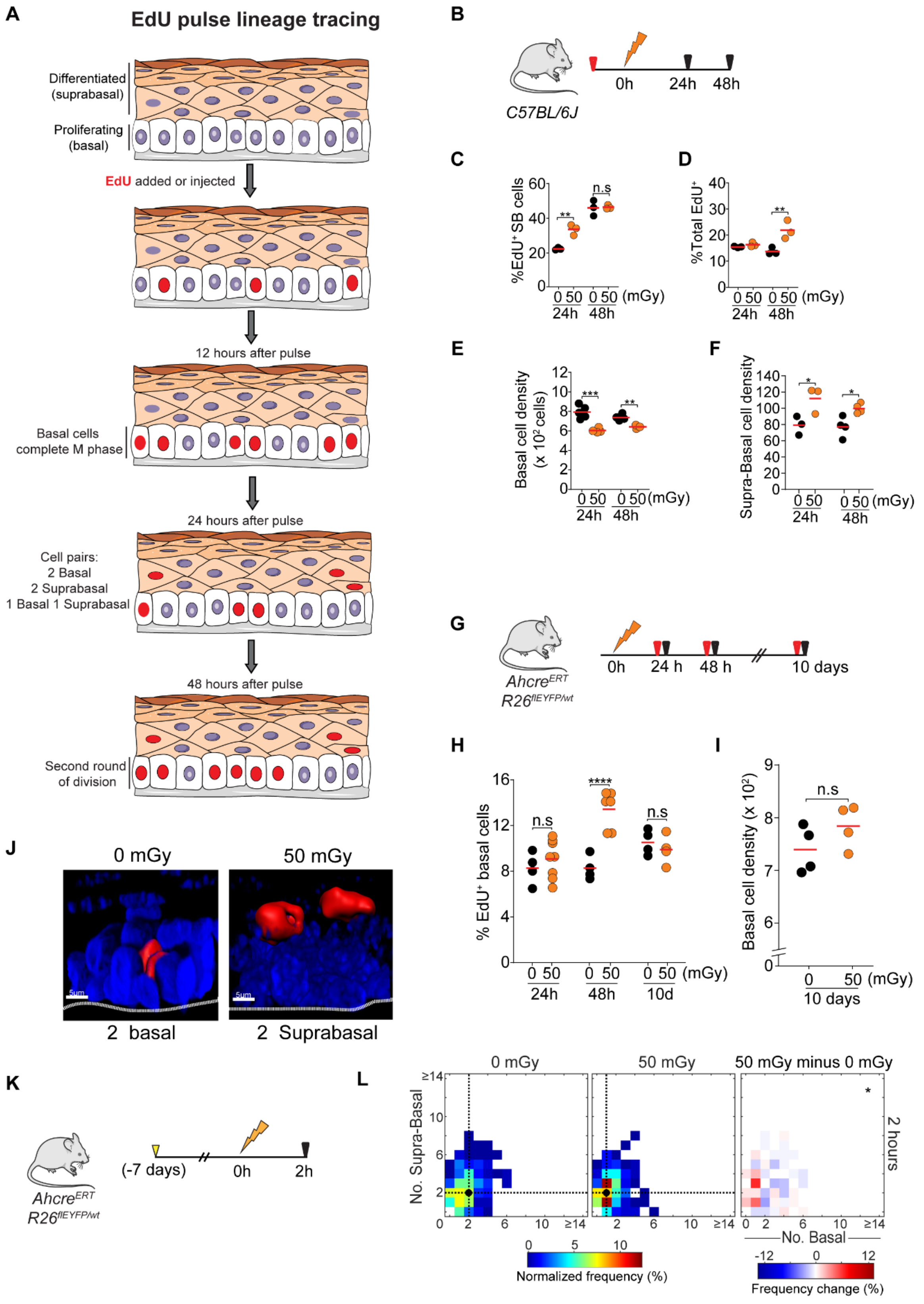
A
EdU pulse lineage tracing
Differentiated (suprabasal)
Proliferating (basal)
EdU added or injected
12 hours after pulse
Basal cells complete M phase
24 hours after pulse
Cell pairs:
2 Basal
2 Suprabasal
1 Basal 1 Suprabasal
48 hours after pulse
Second round of division
B
C57BL/6J
0h 24h 48h
C
%EdU+ SB cells
D
%Total EdU+
E
Basal cell density (x 10² cells)
F
Supra-Basal cell density
(mGy)
24h 48h
G
AhcreERT R26flEYFP/wt
0h 24 h 48 h 10 days
H
% EdU+ basal cells
24h 48h 10d
I
Basal cell density (x 10²)
10 days
n.s
J
0 mGy
50 mGy
2 basal
2 Suprabasal
K
AhcreERT R26flEYFP/wt
(-7 days) 0h 2h
L
0 mGy
50 mGy
50 mGy minus 0 mGy
No. Supra-Basal
No. Basal
2 hours
Normalized frequency (%)
Frequency change (%)

Figure S3. EdU lineage tracing following low dose radiation. Related to Figure 2.

(A) EdU pulse lineage cell tracing. Cartoon shows where EE EdU labelled cells (red) are located at different time points after EdU labelling. (B) Experimental protocol. Mice were given EdU 1 hour before irradiation (red triangle) to allow number and location of EdU^+ cells in EE to be tracked by imaging 24 and 48 hours post irradiation. (C-F) Percentage of EdU^+ suprabasal (C) EdU^+ total cells (D) basal cell density (E) and supra-basal cell density (F) 24 and 48 hours after 0 or 50 mGy LDIR. Points are mean values from individual mice. n=3, 4 or more mice per condition **** p<0.0001, *** p<0.001, **p<0.01, *p<0.05, n.s. not significant (unpaired t test). Total cells per condition in (C) was >4000, >6000 in (D) and (E) and >3500 basal cells in (F). (G) Experimental protocol. *Cyp1A1cre^{ERT}Rosa26$^{flEYFP/wt}$* mice were irradiated with 0 or 50 mGy LDIR (black arrows) and were given EdU (red arrows) 1 hour before culling for each time point. EdU^+ basal cells were detected by confocal imaging. (H) Percentage of EdU^+ basal cells at time points shown in (G) ****p<0.0001, n.s. not significant, (unpaired t test). (I) Basal cell density (as number of cells per field of view) 10 days after 0 or 50 mGy LDIR. At least 2900 basal cells were quantified per condition in 4 mice per condition. (J) Rendered confocal z stacks showing side views of typical EdU^+ clones found after LDIR, containing two basal cells (for 0 mGy) and two suprabasal cells (for 50 mGy). EdU^+ (red), DAPI (blue). Dashed lines indicate basement membrane, scale bars, 5 µm. (K) Experimental protocol: *cre* was induced 7 days before irradiation in *Cyp1A1cre^{ERT}Rosa26$^{flEYFP/wt}$* reporter mice (RYFP), yellow arrow. Samples were collected at 2 hours post irradiation and clones size were analyzed. (L) Heat maps representing frequency of clones with the number of basal and supra-basal cells indicated and differences between 0 and 50 mGy irradiated animals (right hand panel) at 2 hours. Black dots and dashed lines indicate geometric median clone-size. *p = 0.042 (Peacock's test). n = 300 clones per condition.

Figure S4

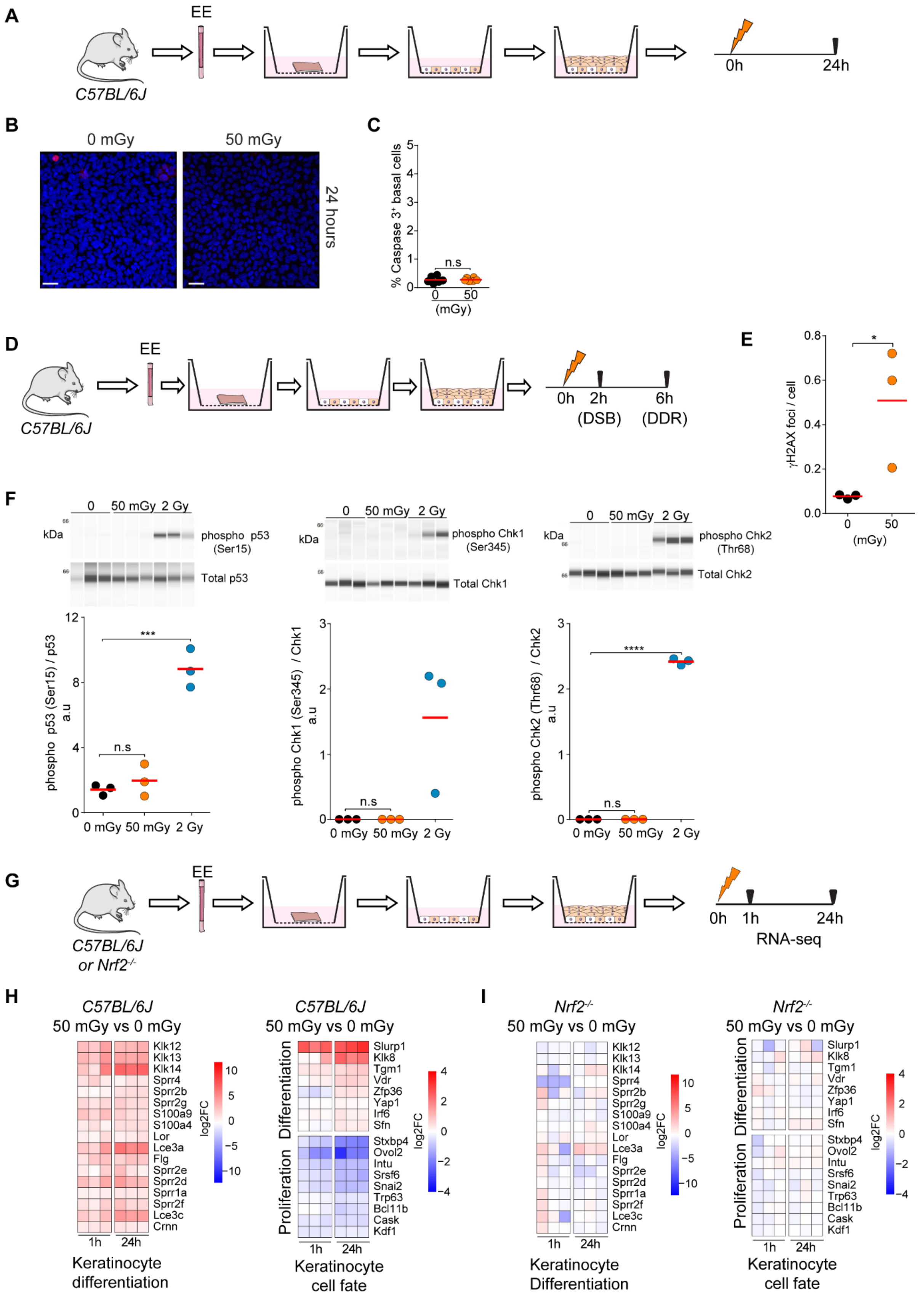

A
EE
C57BL/6J
0h
24h
B
0 mGy
50 mGy
24 hours
C
% Caspase 3⁺ basal cells
n.s
0
50
(mGy)
D
EE
C57BL/6J
0h
2h
6h
(DSB)
(DDR)
E
γH2AX foci / cell
*
0
50
(mGy)
F
0
50 mGy
2 Gy
kDa
phospho p53 (Ser15)
Total p53
phospho Chk1 (Ser345)
Total Chk1
phospho Chk2 (Thr68)
Total Chk2
phospho p53 (Ser15) / p53 a.u
phospho Chk1 (Ser345) / Chk1 a.u
phospho Chk2 (Thr68) / Chk2 a.u
***
****
n.s
0 mGy
50 mGy
2 Gy
G
EE
C57BL/6J or Nrf2-/-
0h
1h
24h
RNA-seq
H
C57BL/6J
50 mGy vs 0 mGy
Klk12
Klk13
Klk14
Sprr4
Sprr2b
Sprr2g
S100a9
S100a4
Lor
Lce3a
Flg
Sprr2e
Sprr2d
Sprr1a
Sprr2f
Lce3c
Crnn
log2FC
1h
24h
Keratinocyte differentiation
Differentiation
Proliferation
Slurp1
Klk8
Tgm1
Vdr
Zfp36
Yap1
Irf6
Sfn
Stxbp4
Ovol2
Intu
Srsf6
Snai2
Trp63
Bcl11b
Cask
Kdf1
Keratinocyte cell fate
I
Nrf2-/-
50 mGy vs 0 mGy
Keratinocyte Differentiation
Keratinocyte cell fate

Figure S4. Effects of 50mGy irradiation on primary esophageal 3D cultures. Related to Figures 2 and 3.

(A) Experimental protocol. Primary mouse keratinocyte 3D cultures were irradiated with 0 or 50 mGy LDIR and analyzed for Caspase3$^+$ basal cells 24 hours later. (B) Top down views of confocal z stacks of typical *in vitro* primary keratinocyte 3D cultures from *C57BL/6J* mice stained for Caspase 3$^+$ (red). DAPI (blue). Scale bars, 30 µm. (C) Percentage of Caspase 3$^+$ basal cells in (B). n.s not significant (unpaired t test). n=6 mice. (D) Experimental protocol. Cultures were irradiated with 0 or 50 mGy LDIR or 2Gy and stained with the double strand DNA break marker γH2AX, 2 hours after irradiation. Staining visualized by confocal microscopy. (E) Number of γH2AX foci per cell in primary keratinocyte 3D cultures from (D) * p<0.05 (unpaired t-test). n= 3 mice per condition. At least 4000 basal cells per condition were quantified. (F) Quantitative immuno-capillary electrophoresis analysis of DNA damage response (DDR) pathway key phosphorylation events (phospho p53 Ser15, phospho CHK1 Ser345 and phospho CHK2 Thr68) for primary keratinocyte 3D cultures shown in (D) 6 hours after irradiation. 2 Gy irradiated samples were included as positive control of DDR pathway phosphorylation. n.s. not significant, **** p<0.0001, *** p<0.001 (unpaired t-test). n= 3 mice per condition. (G) Experimental protocol. 3D cultures from *C57BL/6J* and *Nrf2*$^{-/-}$ animals were irradiated with 0 or 50 mGy LDIR and total RNA was isolated from samples 1 hour and 24 hours after irradiation. (H and I) RNA-seq generated heat maps, performed on biological triplicate cultures, 1 and 24 hours after 50 mGy LDIR compared to 0 mGy, for *C57BL/6J* (H) and *Nrf2*$^{-/-}$ (I) focusing on transcripts with adjusted p<0.05, involved in keratinocyte differentiation (first panel), and keratinocyte cell fate regulation (second panel).

Figure S5

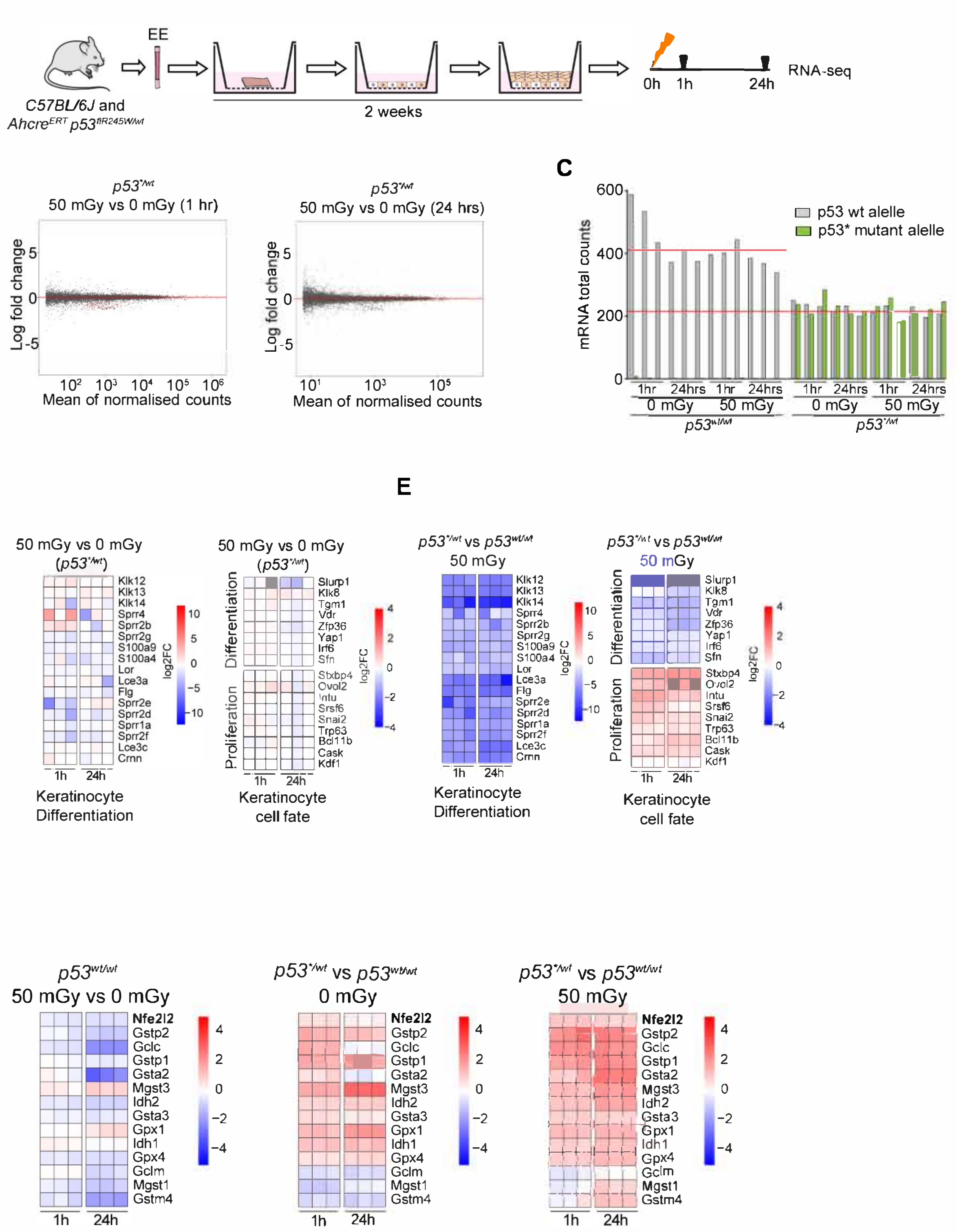
A
EE
C57BL/6J and
AhcreERT p53flR245W/wt
2 weeks
0h 1h 24h
RNA-seq
B
p53*/wt
50 mGy vs 0 mGy (1 hr)
Log fold change
Mean of normalised counts
p53*/wt
50 mGy vs 0 mGy (24 hrs)
Log fold change
Mean of normalised counts
C
mRNA total counts
p53 wt allele
p53* mutant allele
1hr 24hrs 1hr 24hrs
0 mGy 50 mGy
p53wt/wt
p53*/wt
D
50 mGy vs 0 mGy
(p53*/wt)
Klk12
Klk13
Klk14
Sprr4
Sprr2b
Sprr2g
S100a9
S100a4
Lor
Lce3a
Flg
Sprr2e
Sprr2d
Sprr1a
Sprr2f
Lce3c
Crnn
log2FC
1h 24h
Keratinocyte
Differentiation
50 mGy vs 0 mGy
(p53*/wt)
Differentiation
Proliferation
Slurp1
Klk8
Tgm1
Vdr
Zfp36
Yap1
Irf6
Sfn
Stxbp4
Ovol2
Intu
Srsf6
Snai2
Trp63
Bcl11b
Cask
Kdf1
log2FC
Keratinocyte
cell fate
E
p53*/wt vs p53wt/wt
50 mGy
Keratinocyte
Differentiation
p53*/wt vs p53wt/wt
50 mGy
Keratinocyte
cell fate
F
p53wt/wt
50 mGy vs 0 mGy
Nfe2l2
Gstp2
Gclc
Gstp1
Gsta2
Mgst3
Idh2
Gsta3
Gpx1
Idh1
Gpx4
Gclm
Mgst1
Gstm4
1h 24h
p53*/wt vs p53wt/wt
0 mGy
p53*/wt vs p53wt/wt
50 mGy

Figure S5. Transcriptional profile of *p53*$^{*/wt}$ primary esophageal 3D cultures after 50mGy LDIR. Related to Figure 5.

(A) Protocol. Primary keratinocyte 3D cultures from *C57BL/6J* (*p53*$^{wt/wt}$) and *p53*$^{R245W\text{-}GFP/wt}$ (*p53*$^{*/wt}$) mice were irradiated with 0 or 50 mGy LDIR and total RNA was isolated from samples 1 hour and 24 hours after irradiation. (B) MA plots of RNA-seq data of cultures from *p53*$^{*/wt}$ mice, comparing irradiated and unirradiated cultures at times shown, red indicates transcripts with adjusted $p<0.05$. (C) mRNA total counts (expression levels) from RNA-seq data for *p53* alleles in induced wild-type or *p53*$^{*/wt}$ cultures. Bars indicate counts for *p53* wild type (grey) and *p53** (green) mRNAs, red lines show mean for each allele. (D and E) Heat maps from RNA-seq performed on *p53*$^{*/wt}$ biological triplicate cultures, 1 and 24 hours after 50 mGy LDIR compared with 0 mGy (D), and *p53*$^{*/wt}$ versus *p53*$^{wt/wt}$ after 50 mGy LDIR (E) focusing on transcripts with adjusted $p<0.05$, involved in keratinocyte differentiation and keratinocyte cell fate regulation. (F) RNA-seq generated heat maps, performed on *p53*$^{wt/wt}$ and *p53*$^{wt/*}$ 3D cultures showing transcript expression differences with adjusted $p<0.05$ for the indicated comparisons, directly regulated by *Nrf2* (*Nfe2l2*).

Figure S6

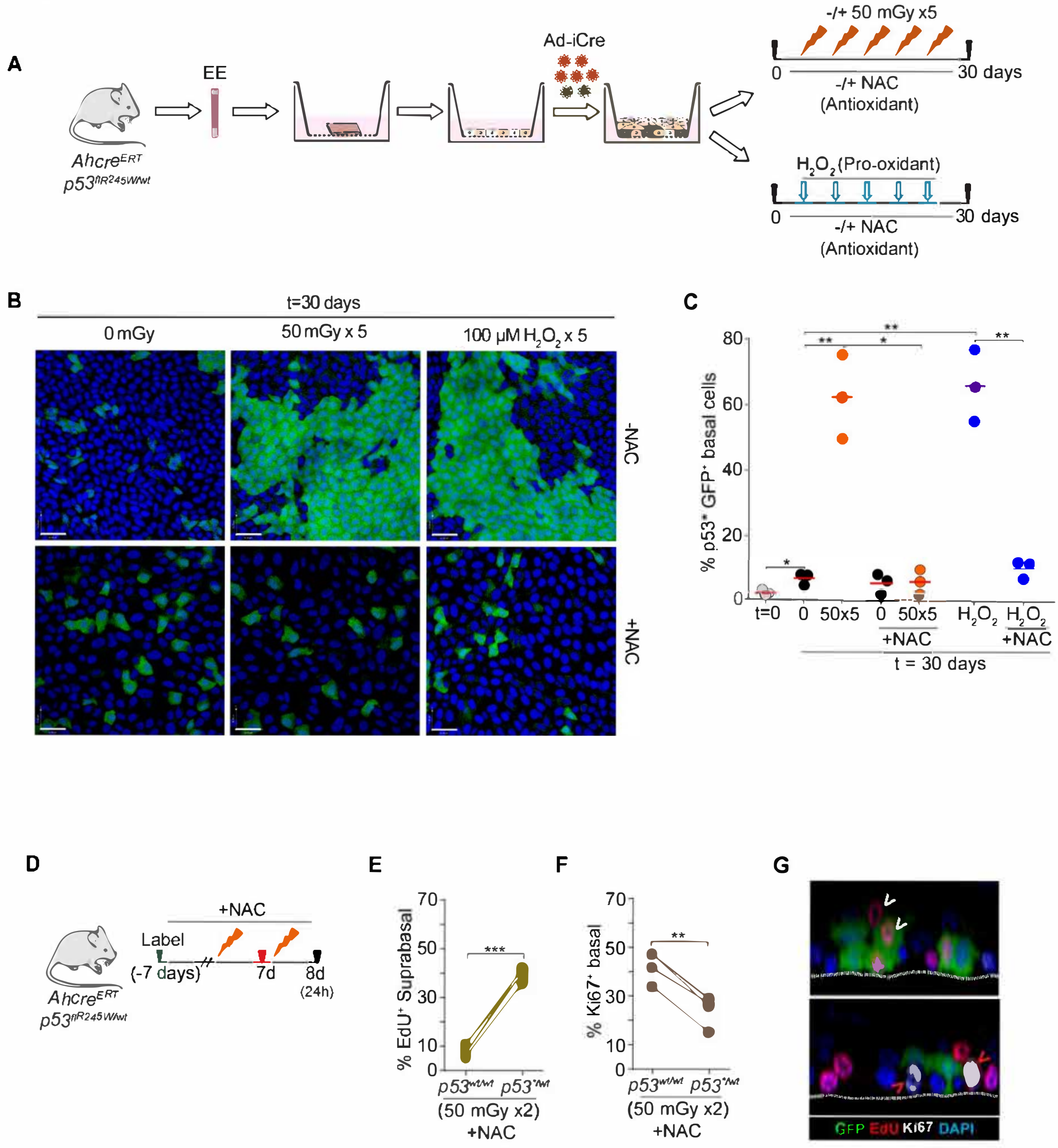

A
AhcreERT p53flR245W/wt
EE
Ad-iCre
-/+ 50 mGy x5
0
30 days
-/+ NAC
(Antioxidant)
H2O2 (Pro-oxidant)
B
t=30 days
0 mGy
50 mGy x 5
100 µM H2O2 x 5
-NAC
+NAC
C
% p53* GFP+ basal cells
t=0
0
50x5
+NAC
H2O2
t = 30 days
D
+NAC
Label
(-7 days)
7d
8d
(24h)
E
% EdU+ Suprabasal
p53wt/wt
p53*/wt
(50 mGy x2)
F
% Ki67+ basal
G
GFP EdU Ki67 DAPI

Figure S6. Effects of 50mGy LDIR and antioxidant treatment on p53*/wt and p53wt/wt in vitro and in vivo. Related to Figure 6.

(A) Experimental protocol. Primary keratinocyte 3D cultures from *p53*$^{R245W\text{-}GFP/wt}$ mice were induced at clonal level by adenoviral cre infection and exposed to five doses of 50 mGy LDIR or five doses of 100µM H_2O_2 with or without NAC treatment. (B) Top down views of confocal z stacks of primary 3D cultures described in (A) showing p53$^{*/wt}$ clones (green), 30 days after different exposures and treatments. Basal layer cells are shown. DAPI (blue). Scale bars, 36 µm.

(C) Percentage of *p53*$^{*/wt}$ basal cells shown in (B). ** p<0.01, * p<0.05, n.s p>0.05 (unpaired t-test). At least 2900 basal cells were quantified per condition. n=3 (independent cultures from different mice). (D) *p53*$^{R245W\text{-}GFP/wt}$ mice (*p53*/wt*) were induced to give expression of p53* in single cells. 7 days later, animals were irradiated with a course of 2 doses of 50 mGy LDIR over 2 weeks. (E and F) Percentage of EdU+ suprabasal cells, (E) and Ki67+ basal cells (F) 24 and 48 hours after 2 doses of 50 mGy in the presence of NAC, in *p53*$^{wt/wt}$ and *p53*$^{*/wt}$ areas within the same animal. Lines link same mouse. ***p<0.001, ** p<0.01 (paired t-test). n= 4 mice per time point. At least 28000 basal cells were analyzed for each time point.

(G) Representative sections of EE whole mounts from p53$^{*/wt}$ mice, 48 hours after 50 mGy LDIR in the presence of NAC, showing EdU+ suprabasal cells (red, marked with white arrows) in *p53** clones and Ki67+ basal cells (white, marked with red arrows) in adjacent *p53*$^{wt/wt}$ cells.

Supplementary Tables and Data

Table S1: Related to Figures 3 and Figure S5.

Fig. 3
Number of quantified mitochondria
(see also data shown in Table S4).

WT	0 mGy	50 mGy
5 min	238521	114768
30 min	197460	105705
60 min	52204	55347
DTT	47347	
H2O2	75413	

WT	0 mGy	0 mGy +DTT	50 mGy	50 mGy+DTT
5 min	63477	50067	66319	66253
30 min	48204	56192	64836	68113
60 min	68880	43877	45182	62252

Fig.S5
Number of quantified mitochondria
(see also data shown in Table S3).

	0 mGy		50 mGy	
	p53$^{wt/wt}$	*p53*$^{*/wt}$	*p53*$^{wt/wt}$	*p53*$^{*/wt}$
5 min	238521	43491	114768	35301
30 min	197450	103688	105705	14730
60 min	52204	43243	55347	7171
DTT	47347	30346		
H2O2	75413	18598		

Table S2: Related to Figures 2, 4-6 and Figure S3.

Number of quantified clones per condition per time point

N= Number of mice per condition per time point

Figure 2D		
	0 mGy	50 mGy
24h	1000	1000
48h	1530	1800

N=8

Figure 4E		
	0 mGy	50 mGy
24h	600	600
48h	600	600

N=4

Figure 5C		
	0 mGy	50 mGy x1
48h	150	450

N=4

Figure 5H and 6E		
	0 mGy	50 mGy x5
-NAC	300	300
+NAC	300	300

N=3

Figure S3L		
	0 mGy	50 mGy x1
2 hours	300	300

N=3
