# Supplementary material for: Outcompeting p53-Mutant Cells in the Normal Esophagus by Redox Manipulation
Source: Cell Stem Cell. 2019 Sep 5;25(3):329–341.e6. doi: 10.1016/j.stem.2019.06.011 (PMC6739485; doi:10.1016/j.stem.2019.06.011)
Supplement: Document S2. Article plus Supplemental Information [file mmc4.pdf]

Cell Stem Cell

Article

Outcompeting *p53*-Mutant Cells in the Normal Esophagus by Redox Manipulation

Graphical Abstract

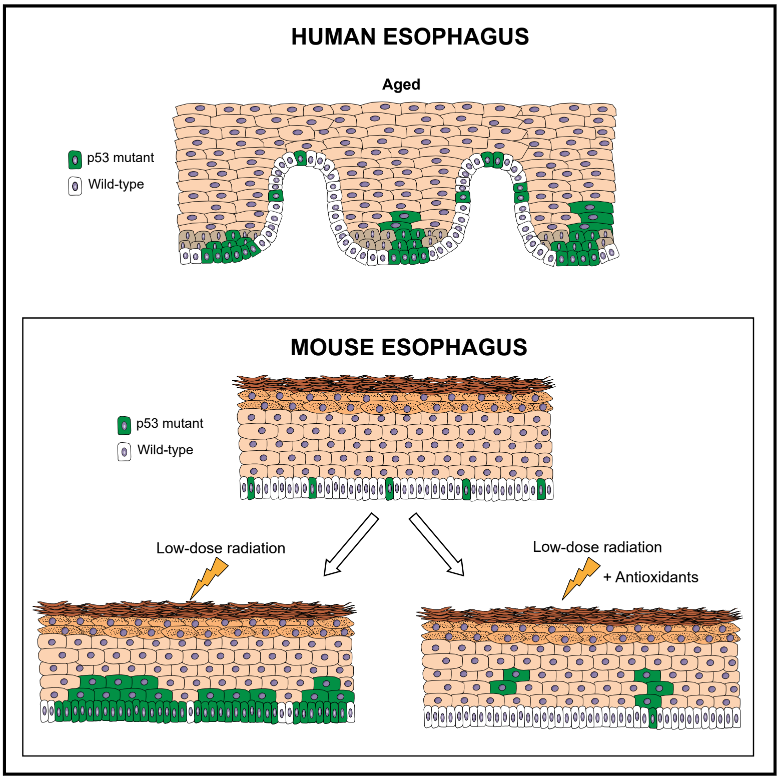

Authors

David Fernandez-Antoran, Gabriel Piedrafita, Kasumi Murai, Swee Hoe Ong, Albert Herms, Christian Frezza, Philip H. Jones

Correspondence

pj3@sanger.ac.uk

In Brief

Normal human esophagus contains *p53* mutant progenitors. Fernandez-Antoran and colleagues show that *p53* mutant progenitors outcompete their wild-type neighbors after low-dose ionizing radiation. This effect is reversed by antioxidant pretreatment. *p53* mutant cells can be displaced from normal tissues via the improvement of the competitive fitness of wild-type progenitors.

Highlights

- Cell tracking shows low-dose ionizing radiation drives differentiation in esophagus
- Low-dose radiation acts via redox stress without activating the DNA repair pathway
- p53 mutant cells do not differentiate after irradiation and outcompete normal cells
- Antioxidant plus radiation depletes p53 mutant cells by improving normal cell fitness

Fernandez-Antoran et al., 2019, Cell Stem Cell *25*, 329–341
September 5, 2019 © 2019 The Author(s). Published by Elsevier Inc.
https://doi.org/10.1016/j.stem.2019.06.011

Cell Stem Cell
Article

CellPress

Outcompeting *p53*-Mutant Cells in the Normal Esophagus by Redox Manipulation

David Fernandez-Antoran,[1] Gabriel Piedrafita,[1,3] Kasumi Murai,[1,3] Swee Hoe Ong,[1] Albert Herms,[1] Christian Frezza,[2] and Philip H. Jones[1,2,4,*]
[1]Wellcome Sanger Institute, Hinxton, Cambridge CB10 1SA, UK
[2]MRC Cancer Unit, University of Cambridge, Box 196, Cambridge Biomedical Campus, Cambridge CB2 0XZ, UK
[3]These authors contributed equally
[4]Lead Contact
*Correspondence: pj3@sanger.ac.uk
https://doi.org/10.1016/j.stem.2019.06.011

SUMMARY

As humans age, normal tissues, such as the esophageal epithelium, become a patchwork of mutant clones. Some mutations are under positive selection, conferring a competitive advantage over wild-type cells. We speculated that altering the selective pressure on mutant cell populations may cause them to expand or contract. We tested this hypothesis by examining the effect of oxidative stress from low-dose ionizing radiation (LDIR) on wild-type and *p53* mutant cells in the transgenic mouse esophagus. We found that LDIR drives wild-type cells to stop proliferating and differentiate. *p53* mutant cells are insensitive to LDIR and outcompete wild-type cells following exposure. Remarkably, combining antioxidant treatment and LDIR reverses this effect, promoting wild-type cell proliferation and *p53* mutant differentiation, reducing the *p53* mutant population. Thus, *p53*-mutant cells can be depleted from the normal esophagus by redox manipulation, showing that external interventions may be used to alter the mutational landscape of an aging tissue.

INTRODUCTION

Normal tissues progressively accumulate cells carrying somatic mutations, some of which are linked to neoplasia and other diseases (Fuster et al., 2017; Martincorena et al., 2015; Suda et al., 2018; Yizhak et al., 2018; Zink et al., 2017). This process is exemplified by human esophageal epithelium (EE), in which mutations generated by cell-intrinsic processes colonize the majority of normal epithelium by middle age (Martincorena et al., 2018; Yokoyama et al., 2019). The most common mutations are under strong positive selection, meaning that there is an excess of protein altering over silent mutations within each gene. This indicates that these mutations confer a competitive advantage over wild-type cells and drive clonal expansions in normal tissue (Alcolea et al., 2014; Alcolea and Jones, 2015; Hall et al., 2018; Murai et al., 2018).

We speculated that, as in other systems of competitive selection, altering the tissue environment may change the relative fitness of particular mutations and their prevalence in the tissue. In this study, we focused on *p53* (human *TP53*, mouse *Trp53*) mutations because these are the most enriched during malignant transformation. *p53* is mutated in 5%–10% of normal EE but in almost all esophageal squamous cell carcinomas (ESCCs) (Martincorena et al., 2018; Cancer Genome Atlas Research Network, 2017). This argues that ESCC emerges from the *p53* mutant cell population in normal epithelium and that mutation of *p53* is required for cancer development.

To investigate the effect of environment on mutational selection, we used mouse EE as a model tissue. This consists of layers of keratinocytes. Proliferation is confined to the lowest, basal cell layer, whereas the upper cell layers contain non-dividing cells that progressively differentiate as they migrate toward the tissue surface, where they are shed (Alcolea et al., 2014; Doupé et al., 2012; Frede et al., 2016; Figure 1A). Although apoptosis is negligible in normal epithelium, cells are continually lost by shedding throughout life, creating a requirement for continuous cell production in the basal layer to maintain cellular homeostasis. This is achieved by a single population of progenitor cells that divide to generate either two progenitor daughters that remain in the basal layer, two differentiated daughters that exit the basal layer, or one cell of each type (Doupé et al., 2012; Marques-Pereira and Leblond, 1965). The outcome of an individual progenitor division is unpredictable, but the probabilities of each outcome are balanced so that, across the population of progenitors, the average cell division generates equal proportions of progenitor and differentiated cells, maintaining cellular homeostasis (Figure 1A).

These insights into normal progenitor cell behavior are key to understanding the dynamics of mutant clones and their selection. Clones carrying neutral mutations that do not alter cell behavior are likely to be lost from the tissue within a few rounds of cell division because, if all progenitor cells divide to produce two differentiated cells, the clone will be lost from the tissue by shedding (Figure 1B). By chance, however, a few neutral mutant clones will expand to a size where the differentiation of all cycling cells within them is unlikely, enabling them to

© 2019 The Author(s). Published by Elsevier Inc.
This is an open access article under the CC BY license (http://creativecommons.org/licenses/by/4.0/).

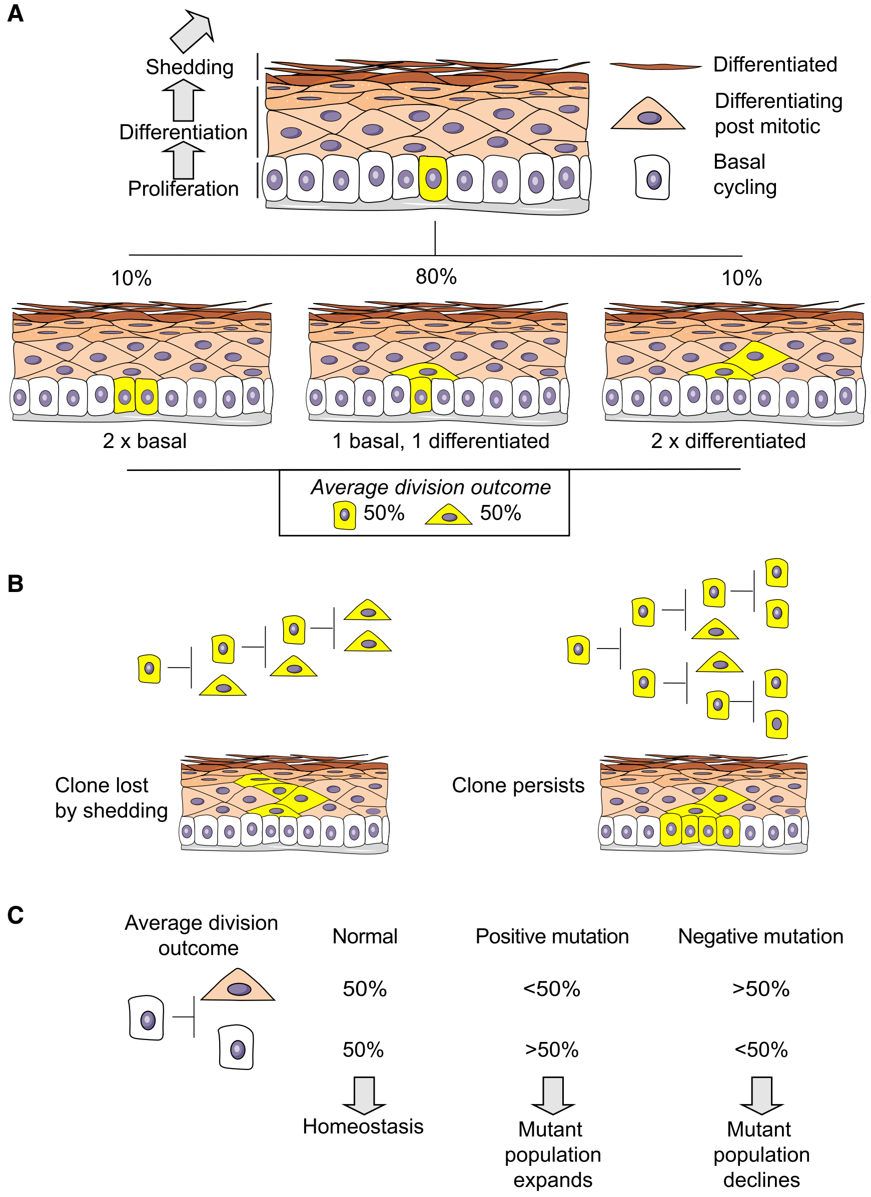

Figure 1. Cell Behavior in Mouse Esophageal Epithelium

(A) Cartoon showing the mouse esophageal epithelium structure. Progenitor cells in the basal layer divide to generate progenitor and differentiating daughter cells. The latter subsequently exit the cell cycle and migrate out of the basal layer through the suprabasal cell layers to the epithelial surface from which they are shed. Progenitor division may generate two progenitors, two differentiated cells, or one of each cell type. The probabilities of each symmetric division outcome (indicated by percentages) are balanced so that, on average, across the basal layer, each division generates 50% progenitors and 50% differentiating cells.

(B) Clonal dynamics. The behavior of progenitors results in most cells that acquire a neutral mutation being lost by differentiation and shedding within a few rounds of division (left clone). Only a few clones will expand to a size that means they are likely to persist long term (right clone).

(C) Positively selected mutants tilt the normally balanced average division outcome toward proliferation, increasing the proportion of persisting mutant clones, whereas a negatively selected mutation that tilts fate toward differentiation will be depleted from the tissue because an increased proportion of clones will be lost by shedding.

persist in the epithelium (Figure 1B; Doupé et al., 2012). Such neutral behavior contrasts with clones harboring positively selected mutations that give the mutant cells a competitive advantage because of the average mutant progenitor division generating more progenitor than differentiated cells (Figure 1C; Alcolea et al., 2014; Frede et al., 2016; Murai et al., 2018). This results in an increased proportion of persistent clones than is seen with neutral mutations (Figure 1C). Furthermore, because there is no restriction of the lateral spread of clones within the basal layer, such clones may expand over a large area (Alcolea et al., 2014; Doupé et al., 2012). Conversely, mutations that tilt the average division outcome toward differentiation have an increased likelihood of loss by shedding and will be outcompeted by wild-type cells.

Insight into normal and mutant cell behavior in EE has come from genetic lineage tracing studies in transgenic mice (Figure S1; Alcolea et al., 2014; Alcolea and Jones, 2013; Doupé et al., 2012; Frede et al., 2016). Here we apply this technique to test whether the selection of *p53* mutant clones is altered by oxidative stress resulting from low-dose ionizing radiation (LDIR) with doses similar to those from medical imaging and environmental contamination (Demb et al., 2017; Hasegawa et al., 2015). We find that LDIR exposure promotes the expansion of *p53* mutant clones. However, when LDIR is combined with antioxidant treatment, wild-type cell fitness exceeds that of the *p53* mutants, which are depleted from the tissue.

RESULTS

LDIR Drives Wild-Type Progenitor Differentiation in EE

We began by determining the effects of a range of whole-body ionizing radiation (IR) doses on EE in wild-type mice. IR exposure led to a dose-dependent increase in double-strand breaks (DSBs) and a decrease in proliferation 24 h after exposure (Figures S2A–S2J). We selected 50 mGy, equivalent to 3–4 computed tomography (CT) scans, for further study (Demb et al., 2017). This dose resulted in an average of one DSB in every 5 cells and did not change proliferation marker expression 24 h after irradiation or induce apoptosis (Figures S2C, S2D, S2G–S2J, and S2K–S2M).

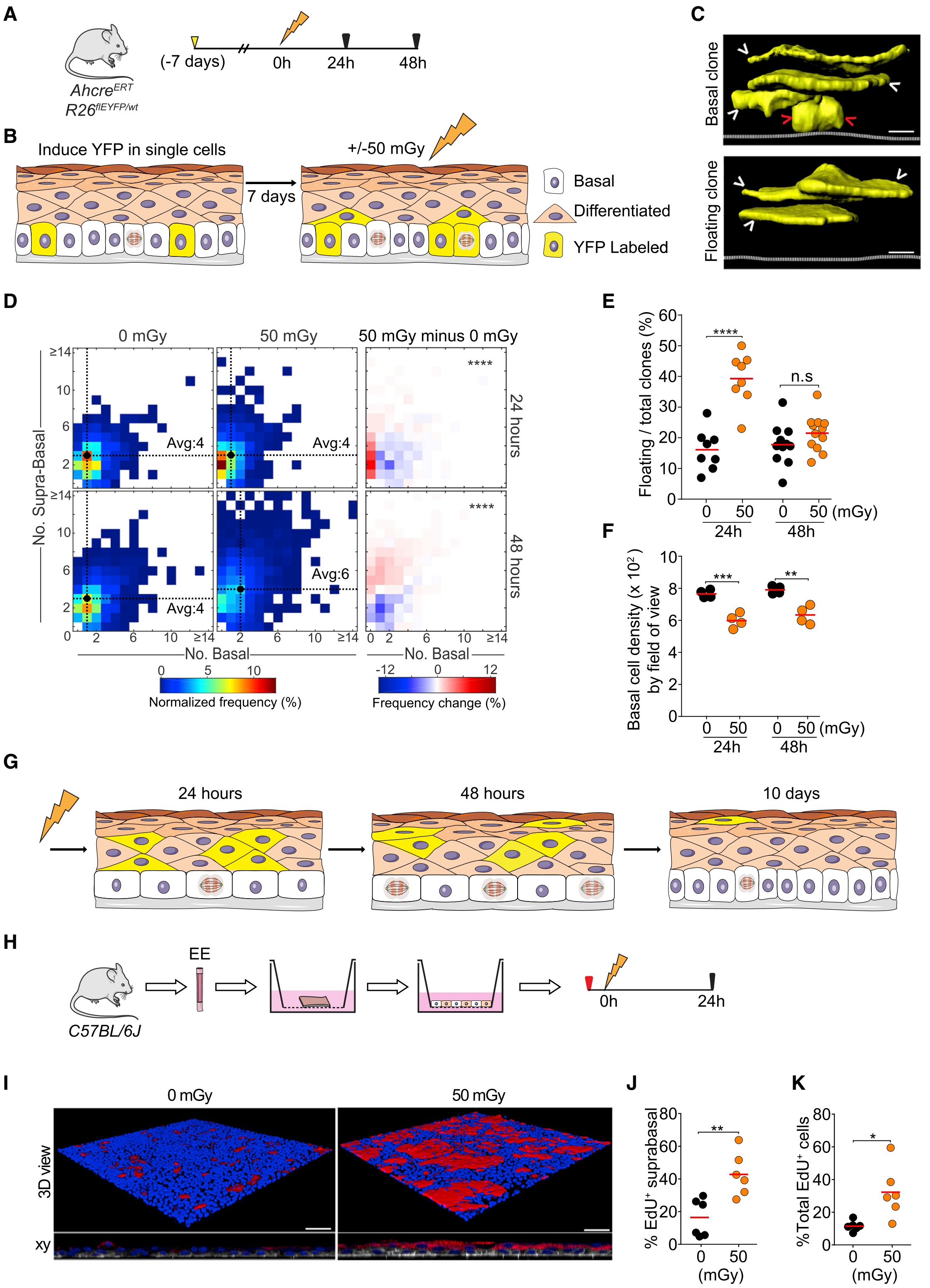

Figure 2. LDIR Promotes Keratinocyte Progenitor Differentiation *In Vivo*

(A) Experimental protocol: *cre* was induced 7 days before irradiation in yellow fluorescent protein (YFP) reporter mice (RYFP, yellow arrow). Samples were collected 24 and 48 h after irradiation.

(B) Lineage cell tracing in EE. YFP is induced in single progenitor cells in the basal cell layer, which generate YFP-expressing clones.

(legend continued on next page)

To track the behavior of esophageal progenitor cells, we used a highly sensitive genetic lineage tracing assay. Yellow fluorescent protein (YFP) expression was induced by genetic recombination in individual progenitor cells in *Ahcre*^{ERT}*Rosa26*$^{flEYFP/WT}$ (*RYFP*) transgenic mice (Figures 2A and S1A). YFP expression was inherited by the progeny of the labeled cell, forming a clone. Analysis of the number and location of cells in multiple clones reveals cell behavior (Alcolea and Jones, 2013; Clayton et al., 2007; Doupé et al., 2012; Figures 2A and 2B). A week after YFP induction, mice were exposed to 50 mGy IR, after which clone sizes were measured by 3D confocal imaging (Figure 2C). 24 h after LDIR, YFP-expressing clones contained a significantly higher proportion of differentiated, suprabasal cells and "floating" clones lacking basal cells destined to be shed than those in non-irradiated animals (Figures 2D and 2E). Basal cell density (cells per area) was reduced, but no radiation-induced apoptosis was detected (Figures 2F and S2K–S2M). These changes were consistent with progenitor cells differentiating and leaving the basal layer over the 24 h after irradiation (Figures 2F, S2E, S2F, S2K–S2M, S3K, and S3L). By 48 h after exposure, the proportion of proliferating basal cells and the size of clones rose (Figures S3H and 2D). These results argue that LDIR induces basal cell differentiation followed by increased compensatory proliferation.

In parallel with genetic lineage tracing, we also performed short-term lineage tracing using ethinyl deoxyuridine (EdU) in animals exposed to LDIR (Frede et al., 2016; Marques-Pereira and Leblond, 1965). A pulse of EdU was used to label the DNA of progenitors in S phase of the cell cycle, allowing us to examine the behavior of a synchronized cohort of proliferating cells. EdU is passed on to the progeny of the labeled cells when they divide, allowing pairs of labeled cells from each division to be visualized by confocal imaging of EE (Figures S3A and S3B). 24 h after 50 mGy irradiation, the proportion of differentiated EdU-labeled cells was significantly increased compared with controls (Figure S3C). There was no change in the total number of EdU cells, indicating no increase in cell division (Figure S3D). The density of cells in the basal layer was reduced in irradiated mice and consistent with an increase in suprabasal cell density unmatched by an increase in proliferation (Figures S3E and S3F). By 48 h after exposure, the total number of EdU$^+$ cells was higher in irradiated mice than in control mice, indicating an increase in cell division compensating for basal cell differentiation.

Consistent with a delayed onset of proliferation, measuring the proportion of basal cells in S phase by giving mice EdU 1 h before culling revealed an increase in S phase cells 48 h after irradiation (Figures S3G and S3H). By 10 days, EE in irradiated mice had returned to homeostasis, with a similar proportion of S phase basal cells and basal cell density as control animals (Figure S3I). We concluded that both genetic and EdU lineage tracing indicate that LDIR alters cell dynamics in EE, inducing a wave of basal cell differentiation followed by increased proliferation that restores the tissue to homeostasis (Figure 2G). A similar delay in cell division following differentiation of nearby cells has also been observed in the epidermis (Mesa et al., 2018).

We then assessed whether the induction of differentiation was a direct effect of LDIR on EE cells or an indirect consequence of irradiating another cell type. To this end, we established primary 3D keratinocyte cultures from explants of murine EE and exposed them to 0 or 50 mGy LDIR (Figure 2H). We observed a substantial increase in the proportion of suprabasal cells expressing the differentiation marker KRT4 24 h after irradiation (Figure 2I). There was no detectable radiation-induced apoptosis (Figures S4A–S4C). EdU lineage tracing results showed increased differentiation and proliferation paralleling the changes 48 h after LDIR exposure *in vivo* (Figures 2J and 2K). This confirms that LDIR acts directly on esophageal keratinocytes to induce differentiation.

Mitochondrial Redox Balance Is Altered after 50 mGy of LDIR in Primary Mouse Keratinocytes

Next we investigated the mechanisms by which LDIR acts on esophageal basal cells using primary 3D keratinocyte cultures. One possibility is activation of the cellular DNA damage response (DDR), which has been shown to promote differentiation in some cell lineages (Sherman et al., 2011). However, despite LDIR generating a number of DSBs similar to that seen *in vivo*, we found no detectable induction of the DNA DDR pathway, as assessed by levels of phosphorylated p53, CHK1, and CHK2 proteins after 50 mGy irradiation (Figures S4D–S4F). In contrast, a 2-Gy dose had a robust effect on DDR protein phosphorylation (Figure S4F). This reveals that LDIR is unlikely to act via the DDR pathway.

An alternative mechanism of inducing differentiation is LDIR-induced oxidative stress because ionizing radiation can result in a prolonged increase in the production of reactive oxygen

(C) Rendered confocal z stacks showing side views of a typical basal clone containing one or more basal cells (top panel) and a floating clone with no basal cells (bottom). Arrowheads indicate basal cells (red) and suprabasal cells (white). Dashed lines indicate the basement membrane. Scale bars, 10 μm.

(D) Heatmaps representing the frequency of clone sizes with the number of basal and supra-basal cells indicated (left panels) and differences between 0 and 50 mGy irradiated animals (right panels) at 24 and 48 h. Black dots and dashed lines indicate the geometric median clone size. Average total clone size is indicated within each plot. ****$p = 6 \times 10^{-8}$ and 5×10^{-48} at 24 and 48 h, respectively (Peacock's test). n = 1000 clones per condition at 24 h and 1,530 (0 mGy) and 1,800 (50 mGy) clones at 48 h.

(E and F) Percentage of floating to total clones (E) and basal cell density (F) 24 and 48 h after irradiation. Each dot is the mean value from one mouse. ****$p < 0.0001$, ***$p < 0.001$, and **$p < 0.01$ (unpaired t test). In (F), at least 12,000 basal cells were analyzed per condition.

(G) Summary. After LDIR, basal cell density decreases, and numerous floating clones appear as cells migrate out of the basal layer. Proliferation then increases, and by 10 days, the epithelium is restored to normal.

(H) Primary EE culture protocol. EE generated from explants was treated with a 1-h pulse of EdU (red arrow) exposed to LDIR and analyzed 24 h later.

(I) Rendered confocal z stacks of typical cultures 24 h after 0 or 50 mGy LDIR. Differentiated suprabasal keratinocytes stain for KRT4 (red), wheat germ agglutinin (white), and DAPI (blue). Scale bars, 25 μm.

(J) and (K) *In vitro* EdU lineage tracing. Shown are the percentage of EdU$^+$ suprabasal cells (J) and percentage of EdU$^+$ total cells (K) after 0 or 50 mGy LDIR. Each point represents the mean from a biological replicate culture from a different mouse. **$p < 0.01$, *$p < 0.05$ (unpaired t test); n = 6; total EdU$^+$ cells, 1,291 (0 mGy), 4,256 (50 mGy).

See also Figures S1–S4 and Tables S2 and S4.

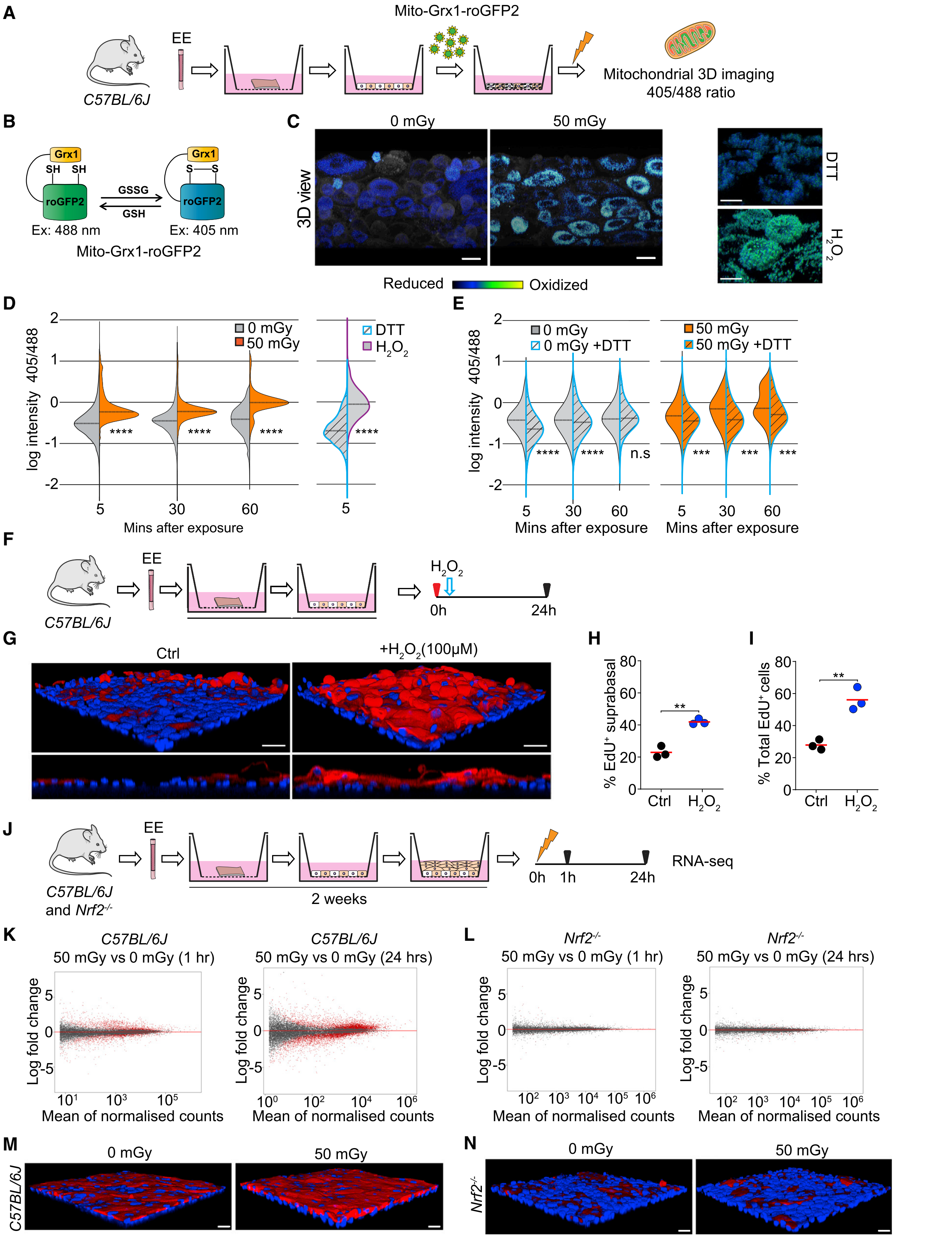

(legend on next page)

species by mitochondria (Azzam et al., 2012; Rodrigues-Moreira et al., 2017). Mitochondria are also key players in the regulation of keratinocyte differentiation (Bhaduri et al., 2015; Hamanaka et al., 2013; Motohashi et al., 2004; Wakabayashi et al., 2003). We speculated that LDIR may act by dysregulating the mitochondrial redox state in esophageal cells. To test this, we introduced a mitochondrially targeted redox-sensitive fluorescent sensor, Mito-Grx1-roGFP, into primary EE cultures (Gutscher et al., 2008; Figures 3A and 3B). The fluorescence characteristics of the sensor changed after exposing the cells to LDIR, indicating increased mitochondrial oxidation (Figures 3C and 3D). These changes were abolished by treating cells with the reducing agent DTT, confirming that they were linked to the mitochondrial oxidation state (Figure 3E). We also treated cells with the oxidant hydrogen peroxide (H_2O_2), finding that a dose that induces similar levels of mitochondrial oxidation as 50 mGy irradiation also drives basal cell differentiation, as assessed by staining for the differentiation marker KRT4 and lineage tracing with EdU (Figures 3F–3I).

The above findings are consistent with LDIR driving differentiation via changes in mitochondrial oxidation. If this is the case, then we might expect cells in which the responses to oxidative stress are disabled because of the lack of the master regulator of the cellular oxidative stress response, the transcription factor *Nrf2* (*Nfe2l2*), to be insensitive to LDIR. We analyzed cultures of wild-type EE using RNA sequencing, finding strong induction of transcripts linked to keratinocyte differentiation within 1 h of exposure to 50 mGy, which became more pronounced 24 h after irradiation (Figures 3J, S4G, and S4H). However, in cultures of EE from *Nrf2*$^{-/-}$ mice, there were no statistically significant changes in transcription following 50 mGy irradiation (Figures 3L and S4I). This suggests that an intact cellular oxidative stress response pathway is required for LDIR-induced differentiation.

Antioxidant Treatment Abolishes NRF2 Nuclear Translocation and Radiation-Induced Differentiation *In Vivo*

Motivated by our observations in cultures, we set out to test whether LDIR acted via oxidative stress *in vivo*. We began by immunostaining EE from irradiated and control mice for phosphoserine40 NRF2 protein, which accumulates in the nucleus of cells following an oxidative challenge (Huang et al., 2002; Jiang et al., 2015). We found nuclear staining in basal layer cells 24 h after LDIR exposure, consistent with increased oxidative stress (Figures 4A and 4B). Next we investigated whether treating mice with the antioxidant N-acetyl cysteine (NAC) would alter the effect of LDIR on EE. NAC treatment at a dose that blocked the accumulation of nuclear NRF2 prevented radiation-induced differentiation of basal cells following 50 mGy IR (Figures 4C–4I).

The *p53*$^{*/WT}$ Mutant Population Expands after Single Exposure or Multiple Exposures to LDIR

Having established that LDIR alters wild-type cell dynamics in EE, we set out to see whether it might influence the selection of cells carrying mutations that drive clonal expansion in the human esophagus. We focused on *p53* as a key a regulator of cellular responses to oxidative and other stresses that is commonly mutated in normal human EE and is almost ubiquitous in ESCC (Martincorena et al., 2018). In addition, both humans and mice with germline heterozygous *p53* mutations are at an increased risk of developing esophageal tumors (Kruiswijk et al., 2015; Shirai et al., 2002). We first investigated the effect of LDIR on *p53* mutant clones in EE using lineage tracing in *Ahcre*^{ERT}*p53*$^{flR245W\text{-}GFPWT}$ transgenic mice, hereafter called *p53*$^{*/WT}$ (Murai et al., 2018). In these animals, *cre*-mediated inducible genetic recombination results in heterozygous expression of the mouse equivalent of the human *TP53*^{R248W} mutant in scattered single cells (Figures S1B and S1C). This *p53* mutant

Figure 3. The Mitochondrial Redox Balance Is Significantly Altered after 50 mGy of LDIR in Primary Mouse Keratinocytes

(A) Experimental protocol. Primary 3D cultures of EE were infected with an adenovirus encoding a genetic sensor of the mitochondrial redox state, irradiated, and imaged.

(B) The Mito-Grx1-roGFP2 reporter is localized to mitochondria. The reduced and oxidized states of the probe are differentially excited by 405 nm and 488 nm light, so the ratio of fluorescence after excitation at the two wavelengths indicates the redox state (Gutscher et al., 2008).

(C) Representative rendered confocal z stacks showing 405 nm/488 nm emission ratios from mitochondria in Mito-Grx1-roGFP2-expressing keratinocytes 60 min after 0 or 50 mGy LDIR, indicated by the pseudo-color scale. Scale bars, 20 μm. Negative (DTT-treated) and positive (H_2O_2-treated) controls for oxidation are shown as well. Scale bars, 15 μm.

(D) Violin plots of the distribution of 405/488 ratios for individual mitochondria in Mito-Grx1-roGFP2 reporter-expressing keratinocytes 5, 30, and 60 min after LDIR, obtained by quantitative confocal 3D imaging. Controls are oxidized (hydrogen peroxide [H_2O_2]-treated) and reduced (DTT-treated) cells ****$p < 0.0001$, ***$p < 0.001$ (Mann-Whitney *U* test). n is the number of mitochondria imaged under each condition, shown in Table S2. Three biological replicate experiments were performed; results from a representative experiment are shown.

(E) Violin plots showing the effect of DTT treatment on irradiated cells. ****$p < 0.0001$, ***$p < 0.001$ (Mann-Whitney *U* test).

(F–I) Experimental protocol.

(F) Primary 3D cultures of EE were labeled with EdU for 1 h and treated with H_2O_2 24 h before immunostaining.

(G) Rendered confocal z stacks of typical cultures 24 h after treatment with the control (Ctrl) or 100 μM H_2O_2. Differentiated suprabasal keratinocytes were stained for KRT4 (red) and DAPI (blue). Dashed lines indicate the insert's membrane. Scale bars, 20 μm.

(H and I) *In vitro* EdU lineage tracing. Shown are the percentage of EdU$^+$ suprabasal cells (H) and percentage of EdU$^+$ total cells (I) after treatment with the Ctrl or 100 μM H_2O_2. Each point represents the mean from a biological replicate culture from a different mouse. **$p < 0.01$ (unpaired t test), n = 3; total EdU$^+$ cells, 2,435 (Ctrl) and 3,256 (H_2O_2).

(J–L) Transcriptional profile of irradiated EE cultures from wild-type *C57BL/6J* and *Nrf2*$^{-/-}$ mice.

(J) Experimental protocol. RNA-seq was performed on biological triplicate cultures 1 and 24 h after 0 or 50 mGy of LDIR.

(K and L) MA plots of RNA-seq data of cultures from *C57BL/6J* (K) and *Nrf2*$^{-/-}$ mice (L) comparing irradiated and unirradiated cultures at the times shown; red indicates differentially expressed transcripts with adjusted $p < 0.05$.

(M and N) Rendered confocal z stacks of *C57BL/6J* (M) and *Nrf2*$^{-/-}$ (N) cultures 24 h after 0 or 50 mGy LDIR. Differentiated suprabasal keratinocytes were stained for KRT4 (red) and DAPI (blue). Scale bars, 25 μm.

See also Figure S4 and Tables S1 and S4.

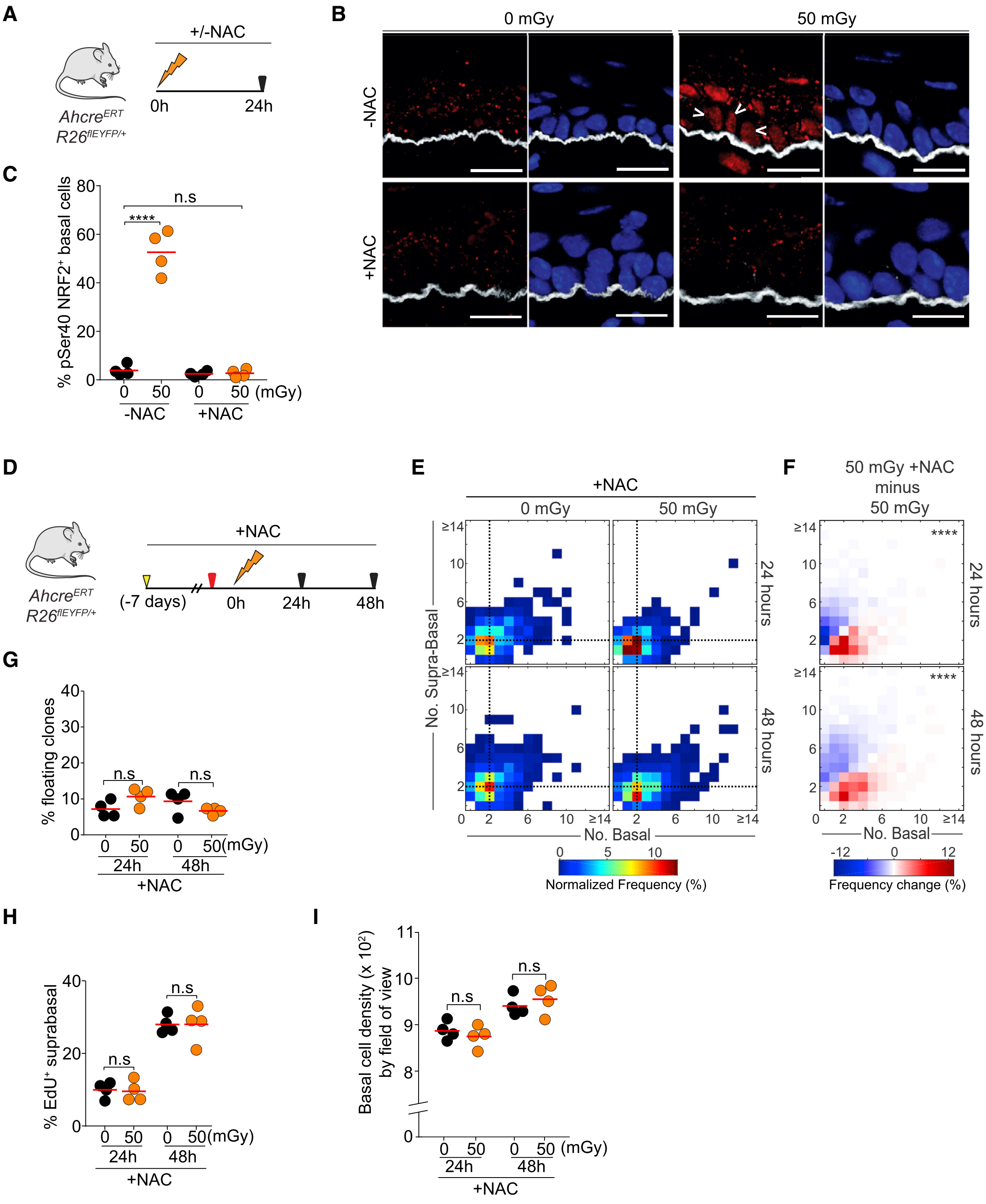

Figure 4. Antioxidant Treatment Abolishes NRF2 Nuclear Translocation and Radiation-Induced Differentiation *In Vivo*

(A) Experimental protocol. YFP reporter mice were exposed to 0 or 50 mGy of LDIR with or without NAC treatment, and EE samples were taken 24 h later.

(B) NRF2 (phospho-serine40) staining (red) in optimal cutting temperature compound (OCT)-embedded, 10-μm-thick cryosections of EE of *Cyp1A1cre*^{ERT} *Rosa26*$^{flEYFP/WT}$ from (A). Arrowheads show basal cell nuclei positive for NRF2. Also shown are the basement membrane marker ITGA6 (white) and DAPI (blue). Scale bars, 14 μm.

(C) Quantification of pSer40 NRF2^{+} basal cells shown in (B). Points are mean values from individual mice. ****$p < 0.0001$; n.s., not significant (unpaired t test); n = 4 mice.

(D) Experimental protocol. YFP reporter mice were given the oral antioxidant N-acetyl cysteine (NAC) 7 days after cell labeling with YFP (yellow arrow) and throughout the experiment, and EdU was injected 1 h prior to LDIR (red arrow). Clone sizes were analyzed 24 and 48 h after irradiation.

(E) Heatmaps showing the frequency of clones containing a number of basal and suprabasal cells observed in 50 mGy versus 0 mGy irradiated animals under NAC treatment. Black dots and dashed lines show geometric median clone size.

(F) Frequency of changes observed in 50 mGy NAC-treated versus 50 mGy non-treated animals. ****$p < 0.0001$ (Peacock's test), n = 600 clones per condition.

(G–I) Percentage of floating clones (G), percentage of EdU^{+} suprabasal cells (H), and basal cell density (I) 24 h and 48 h after 0 or 50 mGy LDIR. Points are mean values from individual mice. n.s., $p > 0.05$ by unpaired t test; n = 4 mice per condition.

See also Tables S2 and S4.

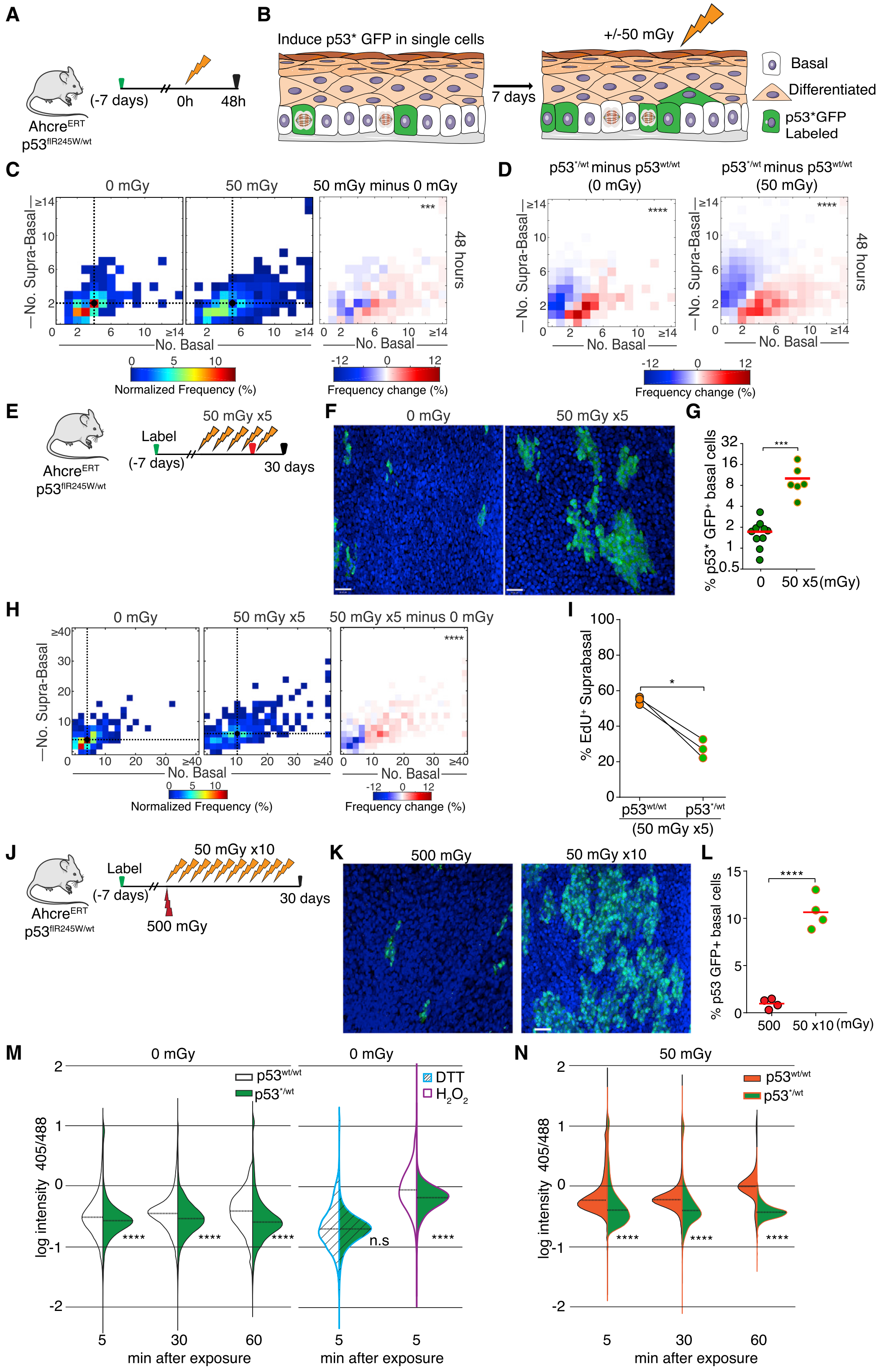

(legend on next page)

has properties distinct from a null allele and is frequently found in keratinocyte-derived cancers (Song et al., 2007; Zerdoumi et al., 2017). In induced $p53^{*/WT}$ mice, *p53**-expressing cells can be tracked because they express a GFP reporter. One week after *p53** induction, animals were exposed to 50 mGy IR (Figures 5A and 5B). We found that $p53^{*/WT}$ clones were significantly larger and had fewer differentiated and more basal cells than both unirradiated $p53^{*/WT}$ mice and $p53^{WT/WT}$ clones in *RYFP* control animals. (Figures 5C and 5D). These changes were more marked after a course of five 50-mGy exposures over 30 days; the size of $p53^{*/WT}$ clones increased substantially, and there was 4-fold increase in the population of p53 mutant basal cells in the epithelium (Figures 5E–5G). The enlarged clones again contained a higher proportion of basal cells than unirradiated controls, pointing to a persistent radiation-induced bias in cell fate from differentiation toward proliferation (Figure 5H). To test this hypothesis, we performed short-term EdU lineage tracing of cells in which a pulse of EdU was delivered prior to the final radiation exposure, allowing the behavior of mutant progenitors to be compared with adjacent unrecombined *p53* wild-type cells in the same animal (Figure 5E). This revealed that cycling p53 mutant progenitors were less likely to differentiate than their wild-type equivalents (Figure 5I). We also compared the effects of a series of ten exposures of 50 mGy LDIR with a single 500-mGy dose. Repeated low doses resulted in a dramatic increase in $p53^{*/WT}$ clone size compared with the single large dose, indicating that the same total dose is more effective at expanding the $p53^{*/WT}$ population when given as multiple small exposures (Figures 5J–5L). This reveals that a single exposure or multiple LDIR exposures act as positive selective pressure for $p53^{*/WT}$ cells in normal EE, based on the resistance of mutant cells to radiation-induced differentiation.

The $p53^{*/WT}$ Mutant Population Is Resistant to Radiation-Induced Differentiation

We next investigated the basis of the competitive advantage of $p53^{*/WT}$ over wild-type cells following LDIR, speculating that the mutant cells may be resistant to the radiation-induced alteration in mitochondrial redox state seen in wild-type cells. In keeping with this hypothesis, primary cultures of $p53^{*/WT}$ EE exhibited lower baseline levels of mitochondrial oxidation than wild-type cells (Figure 5M), and these were little changed in the mutant following exposure to 50 mGy LDIR (Figure 5N). This suggested that $p53^{*/WT}$ may express higher levels of cellular antioxidants, protecting them from the effects of LDIR in a manner similar to NAC treatment of wild-type cells. In keeping with this hypothesis, RNA sequencing (RNA-seq) analysis of mutant cultures revealed minimal change in the global transcriptome or transcription following LDIR (Figures S5A and S5B). We noted that transcription of the *p53** allele was unaltered by LDIR and that, in contrast to wild-type cells, differentiation associated transcripts were not upregulated by irradiation of mutant cultures (Figures S5C–S5E). Both unirradiated and LDIR-treated $p53^{*/WT}$ cells showed substantially higher levels of transcripts encoding oxidative stress response genes compared with $p53^{WT/WT}$ cells (Figure S5F).

To further test whether the competitive advantage of $p53^{*/WT}$ over wild-type cells was mediated by differences in their

Figure 5. The $p53^{*/WT}$ Mutant Population Expands after Single Exposure or Multiple Exposures to LDIR

(A) Experimental protocol. Mice with a conditional $p53^{R245W\text{-}GFP/WT}$ ($p53^{*/WT}$) allele were induced (green arrow), giving p53* and GFP expression in single progenitor cells. 7 days later, animals were irradiated with a single exposure of 50 mGy of LDIR and culled 48 h after the last irradiation.

(B) Cartoon of lineage tracing in this protocol.

(C) Heatmaps showing the frequency of $p53^{*/WT}$ clones containing the number of basal and suprabasal cells indicated (left panels) and the frequency change observed when comparing 0 and 50 mGy irradiated animals (right panel). Black dots, geometric clone-size median. ***p = 0.0007 (Peacock's test); n = 150 and 450, respectively.

(D) Heatmaps showing the frequency change observed when comparing $p53^{*/WT}$ and $p53^{WT/WT}$ (wild-type) clones 48 h after exposure to 0 or 50 mGy LDIR. ****p < 0.0001 (Peacock's test).

(E) Experimental protocol. Mice with a conditional $p53^{R245W\text{-}GFP/WT}$ ($p53^{*/WT}$) allele were induced (green arrow), giving p53* and GFP expression in single progenitor cells. 7 days later, animals were irradiated with five doses of 50 mGy of LDIR over 30 days, commenced with a minimal separation of 3 days between each dose. EdU was given 1 h prior to the last irradiation (red arrow), and animals were culled 48 h later.

(F) Top-down views of confocal z stacks of typical EE whole mounts showing $p53^{*/WT}$ clones (green) after 0 or 50 mGy × 5 LDIR. Basal layer cells are shown (DAPI, blue). Scale bars, 28 μm.

(G) Percentage of $p53^{*/WT}$ basal cells after 0 or 50 mGy × 5. Points show mean values from individual mice. ***p < 0.001 (t test); n = 7 (0 mGy) and n = 6 (50 mGy) mice.

(H) Heatmaps showing the frequency of $p53^{*/WT}$ clones containing the number of basal and suprabasal cells of the indicated sizes (left panels) and the frequency change observed in 0 and 50 mGy × 5 doses irradiated animals (right panel). Black dots and dashed lines indicate geometric median clone size. ****p < 0.0001 (Peacock's test), n = 300 clones per condition.

(I) Comparison of $p53^{*/WT}$ clones with adjacent $p53^{WT/WT}$ EE in the same irradiated animal. Shown is the percentage of EdU^{+} suprabasal cells. Points show mean values from 3 mice, and lines link the same animal. *p < 0.05 (paired t test).

(J) Experimental protocol to study repeated radiation exposure. $p53^{R245W\text{-}GFP/WT}$ mice were induced as in (A) and (E), and 7 days later, animals were irradiated with a single dose of 500 mGy or with a course of ten doses of 50 mGy. At 30 days, both groups were analyzed for $p53^{*/WT}$ clone size.

(K) Top-down views of confocal z stacks of typical EE whole mounts showing $p53^{*/WT}$ clones (green) 1 month after 500 mGy or 50 mGy × 10 LDIR. Basal layer cells are shown (DAPI, blue). Scale bars, 30 μm.

(L) Percentage of $p53^{*/WT}$ basal cells shown in (K). ****p < 0.0001 (t test). At least 25,000 basal cells were quantified per condition. n = 4 mice per condition.

(M) and (N) Primary keratinocyte 3D cultures from $p53^{WT/WT}$ and $p53^{*/WT}$ EE were infected with an adenovirus encoding a genetic sensor of the mitochondrial redox state and irradiated, and single mitochondria were imaged by confocal microscopy. Violin plots show the distribution of 405/488 ratios for individual mitochondria in Mito-Grx1-roGFP2 reporter-expressing keratinocytes from $p53^{WT/WT}$ (white in M; orange in N) and $p53^{*/WT}$ (green) 5, 30, and 60 min after 0 mGy (M) or 50 mGy LDIR (N), obtained by quantitative confocal 3D imaging. Controls are oxidized (H_2O_2-treated) and reduced (DTT-treated) cells for each strain. ****p < 0.0001 (Mann-Whitney *U* test). The numbers of mitochondria imaged under each condition are shown in Table S1. Three biological replicate experiments were performed; results from a representative experiment are shown.

See also Figures S1 and S5 and Tables S2 and S4.

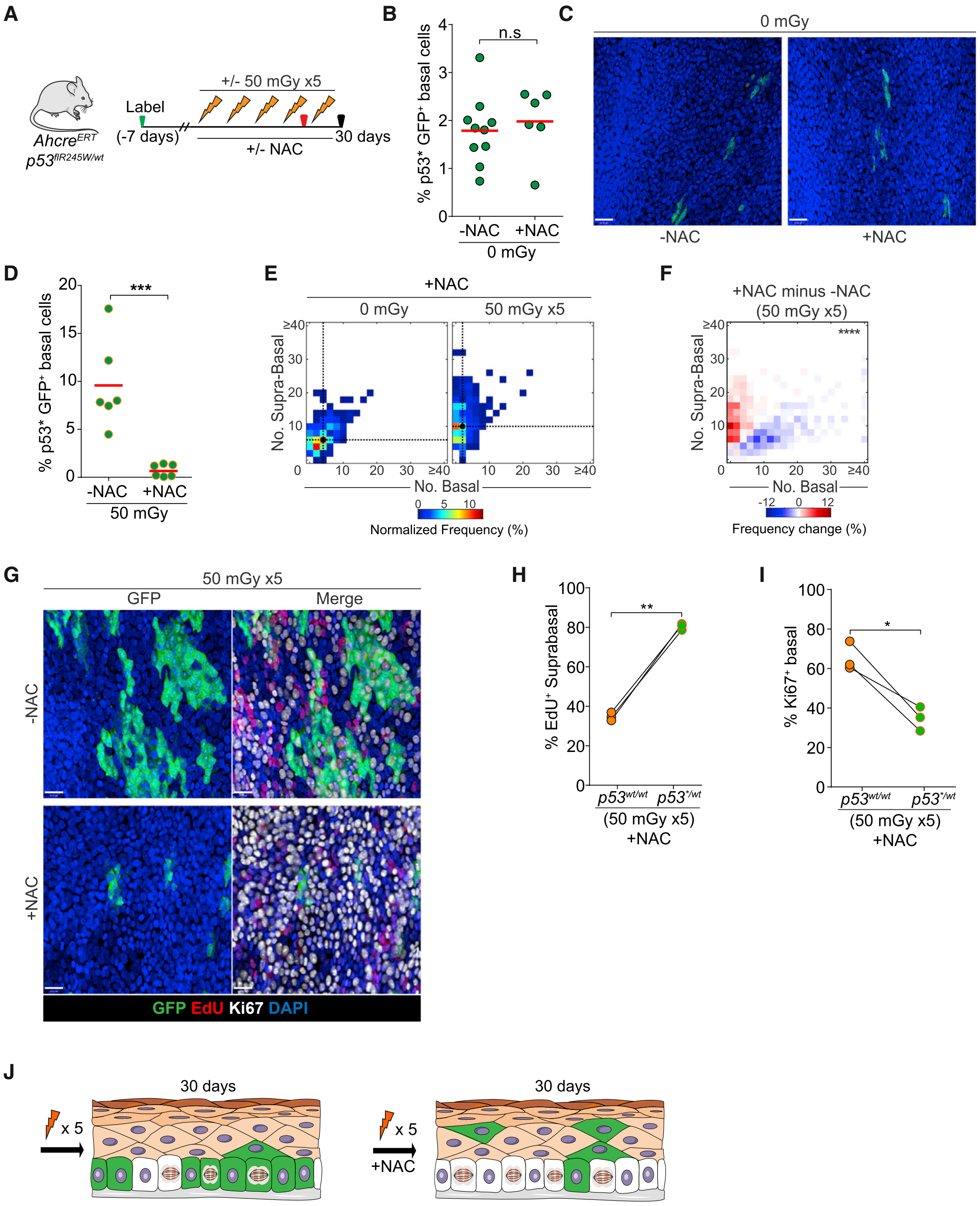

Figure 6. The $p53^{*/WT}$ Mutant Population Decreases after Combined Antioxidant Treatment and Several LDIR Exposures

(A) Experimental protocol. Mice were given the oral antioxidant NAC 7 days after the $p53^{*/WT}$ allele was induced (green arrow) and kept throughout the experiment. Animals were irradiated with five doses of 50 mGy LDIR over 30 days, commenced with a minimal separation of 3 days between each dose. EdU was injected 1 h prior the last irradiation (red arrow).

(B) Percentage of $p53^{*/WT}$ basal cells in mice treated with or without NAC and non-irradiated. Points show mean values from individual mice. n.s., $p > 0.05$ by unpaired t test; n = 10 mice (−NAC) and n = 6 mice (+NAC).

(C) Top-down views of confocal z stacks of typical EE whole mounts showing $p53^{*/WT}$ clones (green) under –NAC and +NAC treatments. Basal layer cells are shown. DAPI is shown in blue. Scale bars, 28 μm.

(D) Percentage of $p53^{*/WT}$ basal cells in mice exposed to 50 mGy × 5 and treated with NAC or left untreated. Points show mean values from individual mice. ***$p < 0.001$ (t test), n = 6 mice per condition.

(E) Heatmaps showing clone sizes in 0 and 50 mGy × 5 irradiated mice treated with NAC. Black dots and dashed lines indicate geometric median clone size.

(legend continued on next page)

response to oxidative stress, we examined mixed cultures containing both *p53* wild-type and mutant cells (Figure S6A). Treatment with LDIR or H_2O_2 resulted in expansion of the mutant population at the expense of wild-type cells (Figures S6B and S6C). The competitive advantage of the p53 mutant cells was abolished by exposing the cultures to NAC.

The *p53*$^{*/WT}$ Mutant Population Decreases after Combined Antioxidant Treatment and Several LDIR Exposures

Taken together, the findings above led us to conclude that the heightened resilience of p53 mutant cells to oxidative stress explains their selection over wild-type cells in EE exposed to LDIR. This motivated us to test whether NAC treatment could also block LDIR-driven mutant clone expansion *in vivo* in *p53*$^{*/WT}$ mice (Figure 6A). We found that NAC treatment alone had no significant effect on the burden of *p53*$^{*/WT}$-induced mutant basal cells in EE (Figures 6B and 6C). Remarkably, however, rather than simply rescuing the effect of LDIR on mutant cell expansion, NAC treatment resulted in a substantial decrease in the proportion of *p53*$^{*/WT}$ mutant basal cells in EE (Figure 6D). This change was accompanied by a significant shift from basal to differentiated cells and an increase in the proportion of floating mutant clones with no remaining basal cells, arguing that mutant clones were being lost by differentiation and shedding (Figures 6E and 6F).

To gain more insight into the cellular mechanisms of p53 mutant clone loss in animals treated with NAC and LDIR, we used short-term lineage tracing with EdU, allowing us to track the fate of proliferating mutant and wild-type cells in the same mouse. We found that, 48 h after EdU labeling, the proportion of differentiated EdU-positive cells was significantly higher in mutant than in wild-type cells (Figures 6G and 6H). Correspondingly, we found that the proportion of basal cells expressing the proliferation-associated antigen Ki67 was reduced in mutant compared with wild-type cells (Figure 6I). These findings were consistent independent of whether EdU labeling was performed after 2 or 5 doses of LDIR (Figures S6D–S6G). We conclude that the loss of competitive fitness of mutant relative to wild-type cells is due to a shift in the outcome of cell division. *p53*$^{*/WT}$ mutant basal cells are less likely to produce proliferating daughters than wild-type cells, resulting in depletion of p53 mutant clones, allowing wild-type cells to recolonize areas of the basal layer previously occupied by *p53*$^{*/WT}$ clones (Figure 6J).

DISCUSSION

These results reveal that external factors can modify clonal selection in normal tissues by altering the competitive balance between mutant and wild-type cells. Redox stress induced by LDIR acts as selective pressure by driving wild-type cells to differentiate. However, high-level expression of endogenous antioxidants in *p53* mutant basal cells protects them against radiation-induced differentiation. Loss of wild-type cells from the basal layer facilitates the expansion of mutant clones. Treating animals with an exogenous antioxidant, NAC, protects wild-type cells from LDIR-induced differentiation and turns the tables on mutant clones, which have an increased likelihood of loss by shedding under these conditions.

It is notable that antioxidant treatment alone has no effect on p53 mutant clones in EE. Indeed, in humans, antioxidants have not only been proven to be ineffective in human cancer prevention studies but may increase all-cause mortality (Bjelakovic et al., 2012); the combination of antioxidant treatment followed by a pro-oxidant challenge might remodel the normal tissue mutational landscape in a beneficial manner. Antioxidants may also mitigate the effects of planned LDIR exposure on *p53* mutant clones. More generally, in light of the observation that some aging tissues are patchworks of mutant clones, our results reveal that external interventions that raise the competitive fitness of wild-type cells above that of mutants may be an attractive option for depleting tissues of potentially harmful mutations.

Finally, our results highlight the potential effect of repeated exposure to small doses of ionizing radiation such as what may be encountered by patients having frequent CT scans. Such low doses may have a negligible mutagenic effect but, by altering the dynamics of normal tissues with a high burden of mutant clones, may modulate the risks of neoplastic and other diseases.

STAR★METHODS

Detailed methods are provided in the online version of this paper and include the following:

- KEY RESOURCES TABLE
- LEAD CONTACT AND MATERIALS AVAILABILITY
- EXPERIMENTAL MODEL AND SUBJECT DETAILS
  - Mice strains and induction of allele
  - Irradiation
  - Lineage tracing
  - Primary keratinocyte 3D culture
  - Adenoviral infections
  - Immunofluorescence
  - EdU lineage tracing
  - Basal cell density
  - RNA isolation and RNA sequencing
  - Immune capillary electrophoresis
  - Mitochondrial redox measurements
- QUANTIFICATION AND STATISTICAL ANALYSIS

(F) Frequency change of NAC-treated versus non-treated mice exposed to 50 mGy × 5 LDIR. ****$p < 0.0001$ (Peacock's test), n = 300 clones per condition.
(G) Top-down views of confocal z stacks of typical EE whole mounts showing *p53*$^{*/WT}$ clones (green), Ki67$^+$ basal cells (white), and EdU$^+$ basal cells (red) after 50 mGy × 5 LDIR without or with NAC treatment. Basal layer cells are shown (DAPI, blue). Scale bars, 40 μm.
(H and I) Comparison of *p53*$^{*/WT}$ clones with adjacent *p53*$^{WT/WT}$ EE in the same irradiated animal treated with NAC. Shown are the percentages of EdU$^+$ suprabasal cells (H) and Ki67$^+$ basal cells (I). Points show mean values from 3 mice, and lines link the same animal. **$p < 0.01$, *$p < 0.05$ (paired t test).
(J) Summary. 30 days after 5 doses of 50 mGy, *p53*$^{*/WT}$ clones have expanded through the tissue, replenishing empty space left by *p53*$^{WT/WT}$ clones when they differentiate. When animals are co-treated with the same 5 doses of 50 mGy plus NAC, *p53*$^{*/WT}$ clones are displaced from the tissue by *p53*$^{WT/WT}$ clones.
See also Figures S1 and S6 and Tables S2 and S4.

CellPress

SUPPLEMENTAL INFORMATION

Supplemental Information can be found online at https://doi.org/10.1016/j.stem.2019.06.011.

ACKNOWLEDGMENTS

We thank Esther Choolun and the staff of the Central Biomedical Resources (University of Cambridge), MRC ARES, and Wellcome Sanger Institute RSF facilities for technical support. This work was supported by funding from the European Union FP7-Euratom-Fission award 323267, Risk, Stem Cells and Tissue Kinetics – Ionising Radiation, a Medical Research Council grant-in-aid (to the MRC Cancer Unit), core grants from the Wellcome Trust (098051 and 296194 to the Wellcome Sanger Institute), and a Cancer Research UK program grant (C609/A17257 to P.H.J.).

AUTHOR CONTRIBUTIONS

D.F.-A. designed, validated, and conducted the experiments with support from K.M. and A.H. and input from P.H.J. and C.F. G.P. analyzed clonal and mitochondrial data. S.H.O. performed bioinformatics analysis and data curation. D.F.-A. and P.H.J. wrote the manuscript with input from C.F.

DECLARATION OF INTERESTS

The authors declare no competing interests.

Received: March 12, 2019
Revised: May 14, 2019
Accepted: June 14, 2019
Published: July 18, 2019

REFERENCES

Alcolea, M.P., and Jones, P.H. (2013). Tracking cells in their native habitat: lineage tracing in epithelial neoplasia. Nat. Rev. Cancer *13*, 161–171.

Alcolea, M.P., and Jones, P.H. (2015). Cell competition: winning out by losing notch. Cell Cycle *14*, 9–17.

Alcolea, M.P., Greulich, P., Wabik, A., Frede, J., Simons, B.D., and Jones, P.H. (2014). Differentiation imbalance in single oesophageal progenitor cells causes clonal immortalization and field change. Nat. Cell Biol. *16*, 615–622.

Anders, S., Pyl, P.T., and Huber, W. (2015). HTSeq–a Python framework to work with high-throughput sequencing data. Bioinformatics *31*, 166–169.

Azzam, E.I., Jay-Gerin, J.P., and Pain, D. (2012). Ionizing radiation-induced metabolic oxidative stress and prolonged cell injury. Cancer Lett. *327*, 48–60.

Bhaduri, A., Ungewickell, A., Boxer, L.D., Lopez-Pajares, V., Zarnegar, B.J., and Khavari, P.A. (2015). Network Analysis Identifies Mitochondrial Regulation of Epidermal Differentiation by MPZL3 and FDXR. Dev. Cell *35*, 444–457.

Bjelakovic, G., Nikolova, D., Gluud, L.L., Simonetti, R.G., and Gluud, C. (2012). Antioxidant supplements for prevention of mortality in healthy participants and patients with various diseases. Cochrane Database Syst. Rev. (3), CD007176.

Cancer Genome Atlas Research Network; Analysis Working Group: Asan University; BC Cancer Agency; Brigham and Women's Hospital; Broad Institute; Brown University; Case Western Reserve University; Dana-Farber Cancer Institute; Duke University, and Greater Poland Cancer Centre, et al. (2017). Integrated genomic characterization of oesophageal carcinoma. Nature *541*, 169–175.

Chan, K., Lu, R., Chang, J.C., and Kan, Y.W. (1996). NRF2, a member of the NFE2 family of transcription factors, is not essential for murine erythropoiesis, growth, and development. Proc. Natl. Acad. Sci. USA. *26*, 13943–13948.

Clayton, E., Doupé, D.P., Klein, A.M., Winton, D.J., Simons, B.D., and Jones, P.H. (2007). A single type of progenitor cell maintains normal epidermis. Nature *446*, 185–189.

Demb, J., Chu, P., Nelson, T., Hall, D., Seibert, A., Lamba, R., Boone, J., Krishnam, M., Cagnon, C., Bostani, M., et al. (2017). Optimizing radiation doses for computed tomography across institutions: Dose auditing and best practices. JAMA Intern. Med. *177*, 810–817.

Dobin, A., Davis, C.A., Schlesinger, F., Drenkow, J., Zaleski, C., Jha, S., Batut, P., Chaisson, M., and Gingeras, T.R. (2013). STAR: ultrafast universal RNA-seq aligner. Bioinformatics *29*, 15–21.

Doupé, D.P., Alcolea, M.P., Roshan, A., Zhang, G., Klein, A.M., Simons, B.D., and Jones, P.H. (2012). A single progenitor population switches behavior to maintain and repair esophageal epithelium. Science *337*, 1091–1093.

Frede, J., Greulich, P., Nagy, T., Simons, B.D., and Jones, P.H. (2016). A single dividing cell population with imbalanced fate drives oesophageal tumour growth. Nat. Cell Biol. *18*, 967–978.

Fuster, J.J., MacLauchlan, S., Zuriaga, M.A., Polackal, M.N., Ostriker, A.C., Chakraborty, R., Wu, C.L., Sano, S., Muralidharan, S., Rius, C., et al. (2017). Clonal hematopoiesis associated with TET2 deficiency accelerates atherosclerosis development in mice. Science *355*, 842–847.

Gutscher, M., Pauleau, A.L., Marty, L., Brach, T., Wabnitz, G.H., Samstag, Y., Meyer, A.J., and Dick, T.P. (2008). Real-time imaging of the intracellular glutathione redox potential. Nat. Methods *5*, 553–559.

Hall, M.W.J., Jones, P.H., and Hall, B.A. (2018). Relating evolutionary selection and mutant clonal dynamics in normal epithelia. bioRxiv. https://doi.org/10.1101/480756.

Hamanaka, R.B., Glasauer, A., Hoover, P., Yang, S., Blatt, H., Mullen, A.R., Getsios, S., Gottardi, C.J., DeBerardinis, R.J., Lavker, R.M., and Chandel, N.S. (2013). Mitochondrial reactive oxygen species promote epidermal differentiation and hair follicle development. Sci. Signal. *6*, ra8.

Hasegawa, A., Tanigawa, K., Ohtsuru, A., Yabe, H., Maeda, M., Shigemura, J., Ohira, T., Tominaga, T., Akashi, M., Hirohashi, N., et al. (2015). Health effects of radiation and other health problems in the aftermath of nuclear accidents, with an emphasis on Fukushima. Lancet *386*, 479–488.

Huang, H.C., Nguyen, T., and Pickett, C.B. (2002). Phosphorylation of Nrf2 at Ser-40 by protein kinase C regulates antioxidant response element-mediated transcription. J. Biol. Chem. *277*, 42769–42774.

Jiang, M., Ku, W.Y., Zhou, Z., Dellon, E.S., Falk, G.W., Nakagawa, H., Wang, M.L., Liu, K., Wang, J., Katzka, D.A., et al. (2015). BMP-driven NRF2 activation in esophageal basal cell differentiation and eosinophilic esophagitis. J. Clin. Invest. *125*, 1557–1568.

Kruiswijk, F., Labuschagne, C.F., and Vousden, K.H. (2015). p53 in survival, death and metabolic health: a lifeguard with a licence to kill. Nat. Rev. Mol. Cell Biol. *16*, 393–405.

Love, M.I., Huber, W., and Anders, S. (2014). Moderated estimation of fold change and dispersion for RNA-seq data with DESeq2. Genome Biol. *15*, 550.

Marques-Pereira, J.P., and Leblond, C.P. (1965). Mitosis and Differentiation in the Stratified Squamous Epithelium of the Rat Esophagus. Am. J. Anat. *117*, 73–87.

Martincorena, I., Roshan, A., Gerstung, M., Ellis, P., Van Loo, P., McLaren, S., Wedge, D.C., Fullam, A., Alexandrov, L.B., Tubio, J.M., et al. (2015). Tumor evolution. High burden and pervasive positive selection of somatic mutations in normal human skin. Science *348*, 880–886.

Martincorena, I., Fowler, J.C., Wabik, A., Lawson, A.R.J., Abascal, F., Hall, M.W.J., Cagan, A., Murai, K., Mahbubani, K., Stratton, M.R., et al. (2018). Somatic mutant clones colonize the human esophagus with age. Science *362*, 911–917.

Mesa, K.R., Kawaguchi, K., Cockburn, K., Gonzalez, D., Boucher, J., Xin, T., Klein, A.M., and Greco, V. (2018). Homeostatic Epidermal Stem Cell Self-Renewal Is Driven by Local Differentiation. Cell Stem Cell *23*, 677–686.e4.

Motohashi, H., Katsuoka, F., Engel, J.D., and Yamamoto, M. (2004). Small Maf proteins serve as transcriptional cofactors for keratinocyte differentiation in the Keap1-Nrf2 regulatory pathway. Proc. Natl. Acad. Sci. USA *101*, 6379–6384.

Murai, K., Skrupskelyte, G., Piedrafita, G., Hall, M., Kostiou, V., Ong, S.H., Nagy, T., Cagan, A., Goulding, D., Klein, A.M., et al. (2018). Epidermal Tissue Adapts to Restrain Progenitors Carrying Clonal p53 Mutations. Cell Stem Cell *23*, 687–699.e8.

Peacock, J.A. (1983). Two-dimensional goodness-of-fit testing in astronomy. Monthly Notices of the Royal Astronomical Society *202*, 615–627.

Rodrigues-Moreira, S., Moreno, S.G., Ghinatti, G., Lewandowski, D., Hoffschir, F., Ferri, F., Gallouet, A.S., Gay, D., Motohashi, H., Yamamoto, M., et al. (2017). Low-Dose Irradiation Promotes Persistent Oxidative Stress and Decreases Self-Renewal in Hematopoietic Stem Cells. Cell Rep. *20*, 3199–3211.

Sherman, M.H., Bassing, C.H., and Teitell, M.A. (2011). Regulation of cell differentiation by the DNA damage response. Trends Cell Biol. *21*, 312–319.

Shirai, N., Tsukamoto, T., Yamamoto, M., Iidaka, T., Sakai, H., Yanai, T., Masegi, T., Donehower, L.A., and Tatematsu, M. (2002). Elevated susceptibility of the p53 knockout mouse esophagus to methyl-N-amylnitrosamine carcinogenesis. Carcinogenesis *23*, 1541–1547.

Song, H., Hollstein, M., and Xu, Y. (2007). p53 gain-of-function cancer mutants induce genetic instability by inactivating ATM. Nat. Cell Biol. *9*, 573–580.

Suda, K., Nakaoka, H., Yoshihara, K., Ishiguro, T., Tamura, R., Mori, Y., Yamawaki, K., Adachi, S., Takahashi, T., Kase, H., et al. (2018). Clonal Expansion and Diversification of Cancer-Associated Mutations in Endometriosis and Normal Endometrium. Cell Rep. *24*, 1777–1789.

Wakabayashi, N., Itoh, K., Wakabayashi, J., Motohashi, H., Noda, S., Takahashi, S., Imakado, S., Kotsuji, T., Otsuka, F., Roop, D.R., et al. (2003). Keap1-null mutation leads to postnatal lethality due to constitutive Nrf2 activation. Nat. Genet. *35*, 238–245.

Yizhak, K., Aguet, F., Kim, J., Hess, J., Kubler, K., Grimsby, J., Frazer, R., Zhang, H., Haradhvala, N., Rosebrock, D., et al. (2018). A comprehensive analysis of RNA sequences reveals macroscopic somatic clonal expansion across normal tissues. bioRxiv. https://doi.org/10.1101/416339.

Yokoyama, A., Kakiuchi, N., Yoshizato, T., Nannya, Y., Suzuki, H., Takeuchi, Y., Shiozawa, Y., Sato, Y., Aoki, K., Kim, S.K., et al. (2019). Age-related remodelling of oesophageal epithelia by mutated cancer drivers. Nature *565*, 312–317.

Yu, G., Wang, L.G., Han, Y., and He, Q.Y. (2012). clusterProfiler: an R package for comparing biological themes among gene clusters. OMICS *16*, 284–287.

Zerdoumi, Y., Lanos, R., Raad, S., Flaman, J.M., Bougeard, G., Frebourg, T., and Tournier, I. (2017). Germline TP53 mutations result into a constitutive defect of p53 DNA binding and transcriptional response to DNA damage. Hum. Mol. Genet. *26*, 2591–2602.

Zink, F., Stacey, S.N., Norddahl, G.L., Frigge, M.L., Magnusson, O.T., Jonsdottir, I., Thorgeirsson, T.E., Sigurdsson, A., Gudjonsson, S.A., Gudmundsson, J., et al. (2017). Clonal hematopoiesis, with and without candidate driver mutations, is common in the elderly. Blood *130*, 742–752.

STAR★METHODS

KEY RESOURCES TABLE

REAGENT or RESOURCE	SOURCE	IDENTIFIER
Antibodies		
Caspase 3	Abcam	Cat# ab2302; RRID: AB_302962
Histone H2A.X (phospho Ser139)	Millipore	Cat# 05-636; RRID:AB_309864
53BP1	Novusbio	Cat# NB100-304; RRID:AB_10003037
GFP/YFP	Thermo Fisher Scientific	Cat# A10262; RRID:AB_2534023
Cytokeratin 4	Abcam	Cat# ab9004; RRID:AB_306932
alpha-Tubulin	Cell signaling	Cat# 2125; RRID:AB_2619646
Ki67	Abcam	Cat# ab15580; RRID:AB_443209
Histone H3 (phospho Ser10)	Abcam	Cat# ab14955; RRID:AB_443110
Nrf2 (phospho Ser40)	Abcam	Cat# ab76026; RRID:AB_1524049
Chk1	Santa Cruz	Cat# sc-8408; RRID:AB_627257
Chk1 (phosphor Ser345)	Cell Signaling	Cat# 2348; RRID:AB_331212
Chk2	Cell Signaling	Cat# 2662; RRID:AB_2080793
Chk2 (phospho Thr68)	Cell Signaling	Cat# 2661; RRID:AB_331479
p53	Vector Laboratories	Cat# VP-P956; RRID: AB_2335917
p53 (phospho Ser15)	Cell signaling	Cat# 9284; RRID: AB_331464
p38 MAPK	Cell signaling	Cat# 9212; RRID:AB_330713
p38 MAPK (phosphor Thr180/Tyr182)	Cell signaling	Cat# 9215; RRID:AB_331762
Alexa Fluor 647 Wheat Germ Agglutinin	Invitrogen	Cat# W32466
Alexa Fluor 647 anti-human/mouse CD49f	BioLegend	Cat# 313610; RRID:AB_493637
Alexa Fluor 488 Donkey Anti-Chicken	Jackson ImmunoResearch	Cat# 703-545-155; RRID:AB_2340375
Alexa Fluor 555 Donkey Anti-Mouse	Thermo Fisher Scientific	Cat# A-31570; RRID:AB_2536180
Alexa Fluor 555 Donkey Anti-Rabitt	Thermo Fisher Scientific	Cat# A-31572; RRID:AB_162543
Alexa Fluor 647 Donkey Anti-Rabitt	Thermo Fisher Scientific	Cat# A-31573; RRID:AB_2536183
Alexa Fluor 488 Donkey Anti-Mouse	Thermo Fisher Scientific	Cat# A-21202; RRID:AB_141607
Bacterial and Virus Strains		
Adeno-CMV-iCre	Vector Laboratories	Cat# 1045
Chemicals, Peptides, and Recombinant Proteins		
β-Napthoflavone	MP Biomedicals	Cat# 156738
Tamoxifen	Sigma Aldrich	Cat# N3633
Fish Skin gelatin	Sigma Aldrich	Cat# G7765
Bovine Serum Albumin	Merk Millipore	Cat# 126575
Donkey serum	Sigma Aldrich	Cat# D9633
N-Ethylmaleimide	Sigma Aldrich	Cat# 04259-5G
Sodium Hidroxide	VWR	Cat# 191373M
N-Acetyl L-Cysteine	LKT Labs	Cat# A0918
Hydrogen Peroxide 30%	Merk Millipore	Cat# 107298
DMEM	Thermo Fisher Scientific	Cat# 11971025
DMEM/F-12	Thermo Fisher Scientific	Cat# 31330
HBSS	GIBCO	Cat#14175-053
Insulin	Sigma Aldrich	Cat# I5500
Adenine	Sigma Aldrich	Cat# A3159
Hydrocortisone	Calbiochem	Cat# 386698
Cholera toxin	Sigma-Aldrich	Cat# C8052
Epidermal growth factor	PeproTech	Cat# 100-15

(*Continued on next page*)

Continued

REAGENT or RESOURCE	SOURCE	IDENTIFIER
Apo-Transferrine	Sigma-Aldrich	Cat# T2036
Fetal calf serum	PAA Laboratories	Cat# A15-041
Penicillin-Streptomycin	Sigma Aldrich	Cat# P0781
Amphotericin B	Sigma Aldrich	Cat# A2942
Protease and Phosphatase inhibitor	Thermo Fisher Scientific	Cat# 78415
40, 6-diamidino-2-phenylindole (DAPI)	Sigma Aldrich	Cat# D9542
Polybrene	Sigma Aldrich	Cat# H9268
Trypsin/EDTA	Sigma Aldrich	Cat# T4174
RIPA buffer	Thermo Fisher Scientific	Cat# 89900
Pierce BCA Protein Assay Kit	Thermo Fisher Scientific	Cat# 23227
Critical Commercial Assays		
Click-iT EdU imaging	Life technologies	Cat# C10086
RNeasy Mini kit	QIAGEN	Cat# 74106
Lipofectamine 2000	Thermo Fisher	Cat# 11668-019
QuickSTART Bradford Dye reagents	BioRAD	Cat# 500-0202
Maxi Prep Endotoxin free kit	QIAGEN	Cat# 12362
Wes Simple	ProteinSimple	Cat# P/N 031-108
QIAamp DNA Micro Kit	QIAGEN	Cat# 56304
Deposited Data		
RNaseq data: $p53^{wt/wt}$	This paper	Wt_Ctrl_1A; ENA: ERS1432686
RNaseq data: $p53^{wt/wt}$	This paper	Wt_Ctrl_1B; ENA: ERS1432689
RNaseq data: $p53^{wt/wt}$	This paper	Wt_Ctrl_1C; ENA: ERS1432688
RNaseq data: $p53^{wt/wt}$	This paper	Wt_Ctrl_24A; ENA: ERS1432703
RNaseq data: $p53^{wt/wt}$	This paper	Wt_Ctrl_24B; ENA: ERS1432704
RNaseq data: $p53^{wt/wt}$	This paper	Wt_Ctrl_24C; ENA: ERS1432705
RNaseq data: $p53^{wt/wt}$	This paper	Wt_50mGy_1A; ENA: ERS1432693
RNaseq data: $p53^{wt/wt}$	This paper	Wt_50mGy_1B; ENA: ERS1432694
RNaseq data: $p53^{wt/wt}$	This paper	Wt_50mGy_1C; ENA: ERS1432690
RNaseq data: $p53^{wt/wt}$	This paper	Wt_50mGy_24A; ENA: ERS1432691
RNaseq data: $p53^{wt/wt}$	This paper	Wt_50mGy_24B; ENA: ERS1432692
RNaseq data: $p53^{wt/wt}$	This paper	Wt_50mGy_24C; ENA: ERS1432695
RNaseq data: $p53^{*/wt}$	This paper	p53_mut_Ctrl_1A; ENA: ERS1432636
RNaseq data: $p53^{*/wt}$	This paper	p53_mut_Ctrl_1B; ENA: ERS1432637
RNaseq data: $p53^{*/wt}$	This paper	p53_mut_Ctrl_1C; ENA: ERS1432634
RNaseq data: $p53^{*/wt}$	This paper	p53_mut_Ctrl_24A; ENA: ERS1432638
RNaseq data: $p53^{*/wt}$	This paper	p53_mut_Ctrl_24B; ENA: ERS1432635
RNaseq data: $p53^{*/wt}$	This paper	p53_mut_Ctrl_24C; ENA: ERS1432639
RNaseq data: $p53^{*/wt}$	This paper	p53_mut_50mGy_1A; ENA: ERS1432640
RNaseq data: $p53^{*/wt}$	This paper	p53_mut_50mGy_1B; ENA: ERS1432641
RNaseq data: $p53^{*/wt}$	This paper	p53_mut_50mGy_1C; ENA: ERS1432642
RNaseq data: $p53^{*/wt}$	This paper	p53_mut_50mGy_24A; ENA: ERS1432643
RNaseq data: $p53^{*/wt}$	This paper	p53_mut_50mGy_24B; ENA: ERS1432647
RNaseq data: $p53^{*/wt}$	This paper	p53_mut_50mGy_24C; ENA: ERS1432644
RNaseq data: $Nrf2^{-/-}$	This paper	NRF2KO_Ctrl_1A; ENA: ERS1432706
RNaseq data: $Nrf2^{-/-}$	This paper	NRF2KO_Ctrl_1B; ENA: ERS1432707
RNaseq data: $Nrf2^{-/-}$	This paper	NRF2KO_Ctrl_1C; ENA: ERS1432708
RNaseq data: $Nrf2^{-/-}$	This paper	NRF2KO_Ctrl_24A; ENA: ERS1432709
RNaseq data: $Nrf2^{-/-}$	This paper	NRF2KO_Ctrl_24B; ENA: ERS1432710
RNaseq data: $Nrf2^{-/-}$	This paper	NRF2KO_Ctrl_24C; ENA: ERS1432711

(*Continued on next page*)

Continued		
REAGENT or RESOURCE	SOURCE	IDENTIFIER
RNaseq data: $Nrf2^{-/-}$	This paper	NRF2KO_50mGy_1A; ENA: ERS1432712
RNaseq data: $Nrf2^{-/-}$	This paper	NRF2KO_50mGy_1B; ENA: ERS1432713
RNaseq data: $Nrf2^{-/-}$	This paper	NRF2KO_50mGy_1C; ENA: ERS1432714
RNaseq data: $Nrf2^{-/-}$	This paper	NRF2KO_50mGy_24A; ENA: ERS1432715
RNaseq data: $Nrf2^{-/-}$	This paper	NRF2KO_50mGy_24B; ENA: ERS1432716
RNaseq data: $Nrf2^{-/-}$	This paper	NRF2KO_50mGy_24C; ENA: ERS1432717
Experimental Models: Cell Lines		
Human derived amphotrophic phoenix cell	ATCC	ATCC CRL-3213
Experimental Models: Organisms/Strains		
Mouse: C57BL/6J	The Jackson Laboratory	JAX: 000664
Mouse: $Nfe2l2^{-/-}$	Chan et al., 1996	JAX: 017009
Mouse: $Ahcre^{ERT}Trp53^{flR245W/wt}$	Murai et al., 2018	N/A
Mouse: $Ahcre^{ERT}Rosa26^{flYFP/wt}$	Clayton et al., 2007	N/A
Recombinant DNA		
pLPCX/mito-roGFP2-Grx1	Addgene	#64977
Software and Algorithms		
LAS X	Leica	N/A
Volocity 6 and 6.3	Perkin Elmer	N/A
Imaris	Bitplane	N/A
GraphPad	Prism 6	N/A
STAR 2.5.3a	Dobin et al., 2013	N/A
HTSeq framework version 0.6.1p1	Anders et al., 2015	N/A
R package: DESeq2	Love et al., 2014	https://bioconductor.org/packages/release/bioc/html/DESeq2.html
R package: pheatmap		https://cran.r-project.org/web/packages/pheatmap/index.html
R package: RColorBrewer		https://cran.r-project.org/web/packages/RColorBrewer/index.html
R package: clusterProfiler	Yu et al., 2012	https://bioconductor.org/packages/release/bioc/html/clusterProfiler.html
R package: org.Mm.eg.db		https://bioconductor.org/packages/release/data/annotation/html/org.Mm.eg.db.html
MATLAB R2016b	MathWorks	N/A
Jupyter & Spyder 3.1 (Python 3)	Python Software Foundation	N/A
Other		
Leica TCS SP8	Leica	N/A
RS225 X-Ray irradiator	Xstrahl	RS225
Caesium source irradiator	Gamma-Service medical GmbH	GSR D1
UNIDOS E universal dosimeter	PTW	N/A
UV-irradiator CL-508M	Uvitec	N/A

LEAD CONTACT AND MATERIALS AVAILABILITY

Requests for reagent and resource sharing should be addressed to the Lead Contact, Philip H. Jones (pj3@sanger.ac.uk) who will fulfil requests.

EXPERIMENTAL MODEL AND SUBJECT DETAILS

Mice strains and induction of allele

All experiments were approved by the local ethical review committees at the UK Medical Research Council (MRC), University of Cambridge and Wellcome Sanger Institute, and conducted according to Home Office project licenses PPL70/7543 and PF4639B40 and

Home Office personal license P14FED054. *AhCreERT-R26*$^{flEYFP/wt}$ experimental mice express YFP from the Rosa 26 locus following *cre* induction. In these mice, transcription of a *cre* mutant estrogen receptor fusion protein (*cre*^{ERT}) is induced by β-naphthoflavone. Tamoxifen is also required for CRE-ERT protein to gain access to the nucleus. *p53*$^{eGFP\text{-}R245W/wt}$ mice express the R245W mutant version of *p53* and can be detected by GFP expression following *cre* induction (Murai et al., 2018). Prior to *cre*-mediated recombination these animals (*p53*$^{*/wt}$) express TRP53 protein from two wild-type alleles. Once the wild-type *Trp53* genomic region is deleted by *cre* both the *Trp53* mutant carrying the R245W mutation and the *eGFP* reporter are transcribed. Animals were induced between 10 and 16 weeks of age. *Nrf2*$^{-/-}$ (*Nfe2l2*^{tm1Ywk}) animals were purchased from the Jackson Laboratory, USA. *C57BL/6J* wild-type mice were also used as indicated. All strains were maintained in a *C57BL/6* background. Where indicated, mice were treated with the antioxidant N-acetyl-Cysteine, NAC (Life Technologies) at 2% (w/v) in drinking water for the duration of the experiment. On average mice consumed ca.1 g/kg NAC per day. Experiments were carried out with male and female animals and no gender specific differences were observed.

Irradiation

In vivo total body irradiation (TBI) was performed using a cesium gamma irradiator (Central of Biomedical Services, University of Cambridge). 40 kg lead plates were used to attenuate the dose rate from 1 Gy/min to 16 mGy/min. Gamma dosimetry was performed by RSP Service Ltd, UK and by Personal Dosimetry Service, Public Health England Centre for Radiation, UK, using thermoluminescent dosimeters to confirm that the delivered doses were accurate. *In vitro* irradiation was performed using an Xstrahl RS225 X-Ray irradiator (Xstrahl, Ltd. UK) with copper and lead filters at 195 KV, 10 mA and 37°C. Xstral dosimetry was performed using UNIDOS E universal dosimeter (PTW, Germany) with an ion chamber detector, corrected for air pressure and temperature values for each experiment.

Lineage tracing

Low frequency expression of YFP and GFP in the mouse esophagus was achieved by inducing animals aged 10–16 weeks with an intraperitoneal dose of 80 mg/kg β-naphthoflavone and 0.25 mg tamoxifen (YFP) and 8 mg/kg β-naphthoflavone and 0.1 mg tamoxifen (GFP). Following induction, between three and eight mice per time point were culled and the esophagus collected. Time points analyzed include 24, 48 hours and 1 month after the first irradiation. Total number of clones quantified for each figure can be found in Table S3. Clones were imaged after immunostaining wholemounts of EE, described below, on an SP8 Leica confocal microscope. The numbers of basal and suprabasal cells in each clone were counted under live acquisition mode. Representative images of clones were produced by rendering confocal z stacks with the following settings: 40X objective with 1.5x digital zoom, optimal pinhole, speed 400 Hz, line average 3, optimal step size, and resolution of 1024 × 1024. Images were reconstructed from optical sections using Volocity 6 software (PerkinElmer) and Imaris (Bitplane). Normalized, clone-size distributions were built for each experimental condition and time point from the observed relative frequencies $f_{m,n}$ of clones of a certain size, containing m basal and n suprabasal cells, resulting in two-dimensional histograms (displayed as heatmaps). A 2D histogram of the residuals or differences observed between conditions in the relative frequencies of each particular clone size (i.e., each cell on the grid) was generated when appropriate.

Primary keratinocyte 3D culture

After removing muscle layer with fine forceps, small pieces of esophageal explants (2 mm^2) were placed onto a transparent ThinCert^{TM} insert (Greiner Bio-One) with the epithelium facing upward and the submucosa stretched over the membrane, dried for 30 minutes at 37°C to ensure attachment and cultured in complete FAD medium (50:50) 4.5 g/L D-Glucose, Pyruvate, L-Glutamine D-MEM (Invitrogen 11971-025): D-MEM/F12 (Invitrogen 31330-038), supplemented with 5 μg/ml insulin (Sigma-Aldrich I5500), 1.8 × 10^{-4} M adenine (Sigma-Aldrich A3159), 0.5 μg/ml hydrocortisone (Calbiochem 386698), 1 x10^{-10} M cholera toxin (Sigma-Aldrich C8052), 10 ng/ml Epidermal Growth Factor (EGF, PeproTech EC Ltd 100-15), 5% fetal calf serum (PAA Laboratories A15-041), 5% Penicillin-Streptomycin (Sigma Aldrich, P0781), 1% Amphotericin B (Sigma Aldrich, A2942) and 5 μg/ml Apo-Transferrine (Sigma-Aldrich T2036). Explants were removed after 7 days once keratinocytes have covered half of the membrane. Media was changed every three days. Cholera toxin, epidermal growth factor and hydrocortisone were removed from the medium two weeks before starting experiments. N-Acetyl L-Cysteine (NAC, LKT Labs, A0918) was dissolved in deionized water (1 M stock solution) adjusted to pH 7.2 and then diluted in cell culture medium to 1 mM final concentration.

Adenoviral infections

To establish mouse primary keratinocyte 3D cultures from *p53*$^{GFP\text{-}R245w/+}$ mice, cells were infected with *Cre*-expressing adenovirus (Ad-CMV-iCre, Vectorbiolabs, #1045 UK). Briefly, cells were incubated with adenovirus-containing medium supplemented with Polybrene (Sigma Aldrich, # H9268) (4 μg/ml) for 24 hours at 37°C, 5% CO_2. Cells were washed and fresh medium was added. Infection rates were > 95%.

Immunofluorescence

For wholemount staining, EE from the middle two thirds of the esophagus was prepared by opening longitudinally, removing the muscle layer and incubating for 1 hour and 30 minutes in 20 mM EDTA-PBS at 37°C. The epithelium was then carefully peeled away from underlying tissue with fine forceps, stretched and fixed in 4% paraformaldehyde in PBS for 30 min. For staining, wholemounts were blocked for 1 hour in blocking buffer (0.5% bovine serum albumin, 0.25% fish skin gelatine, 0.5% Triton X-100 and 10% donkey

serum) in PHEM buffer (60 mM PIPES, 25 mM HEPES, 10 mM EGTA, and 4 mM $MgSO_4 \cdot 7H_20$). Primary and secondary antibodies were incubated overnight using blocking buffer, followed by several washes over 2-3 hours with 0.2% Tween-20 in PHEM buffer. A final overnight incubation with 1 μg/ml DAPI in PHEM buffer was used to stain cell nuclei. For staining conventional tissue sections, optimal cutting temperature compound (OCT) embedded esophageal cryosections of 10-14 μm thickness were fixed with 2% paraformaldehyde for 5 min, blocked in blocking buffer and stained with the respective primary and secondary antibodies for 1 hour at room temperature. Samples were washed with PHEM buffer between incubations. EdU incorporation was detected with Click-iT chemistry kit according to the manufacturer's instructions (Invitrogen) using 647 or 555 Alexa Fluor azides. Confocal images were acquired on a Leica TCS SP8 confocal microscope (objectives × 20 and × 40; optimal pinhole; speed 400 Hz; line average 3; resolution 1024 × 1024) and reconstructed using Volocity 6 image processing software (PerkinElmer) and Imaris (Bitplane). Antibodies used are listed in Table S1.

EdU lineage tracing

For *in vivo* lineage tracing, 10 μg of EdU in PBS was administered by intraperitoneal injection, 1 hour before the last irradiation. Tissues were collected, 2, 24 and 48 hours after injection. EdU-positive basal and suprabasal cells were quantified from a minimum of 10 z stack images (objective × 40; optimal pinhole; speed 400 Hz; line average 3; resolution 1024 × 1024, zoom × 1.5) from wholemounts or 3D cultures. For *in vitro* lineage tracing, 10 μM EdU was added to the culture and incubated for 1 hour at 37°C and 5% CO_2. After EdU incubation, media was changed and cells were irradiated with 50 mGy. Samples were collected at different time points after irradiation.

Basal cell density

Relative cell density was calculated in 0 mGy and 50 mGy irradiated mice at different time points by imaging the basal layer from EE wholemounts or 3D cultures and quantifying the number of $DAPI^+$ basal cells per field in 5-10 random images per animal.

RNA isolation and RNA sequencing

Total RNA was extracted from 3D cultures of mouse primary keratinocytes using RNeasy Micro Kit (QIAGEN, UK), following the manufacturer's recommendations, including on column DNase digestion. Briefly, cells were washed with cold Hank's Balanced Salt Solution-HBSS (GIBCO, UK) and then lysis buffer was added directly to the insert. The integrity of total RNA was determined by Qubit RNA Assay Kit (Invitrogen, UK). For RNA-seq, libraries were prepared in an automated fashion using an Agilent Bravo robot with a KAPA Standard mRNA-Seq Kit (KAPA BIOSYSTEMS). In house adaptors were ligated to 100-300 bp fragments of dsDNA. All the samples were then subjected to 10 PCR cycles using sanger_168 tag set of primers and paired-end sequencing was performed on Illumina HiSeq 2500 with 75 bp read length. Reads were mapped using STAR 2.5.3a, the alignment files were sorted and duplicate-marked using Biobambam2 2.0.54, and the read summarization performed by the htseq-count script from version 0.6.1p1 of the HTSeq framework (Anders et al., 2015; Dobin et al., 2013). Differential gene expression was analyzed using the DESeq2 R package (Love et al., 2014), and the downstream pathway analysis and visualization using R (https://www.R-project.org/) and the packages Pheatmap (https://cran.r-project.org/web/packages/pheatmap/index.html), RColorBrewer (https://cran.r-project.org/web/packages/RColorBrewer/index.html), clusterProfiler (Yu et al., 2012) and org.Mm.eg.db (http://bioconductor.org/packages/release/data/annotation/html/org.Mm.eg.db.html). Differentially-expressed genes are hits reported by DESeq2 with adjusted p value (padj) of less than 0.05. Heatmaps were generated from the ratio of TPM values of the treated sample over the average of the respective control samples.

Immune capillary electrophoresis

For protein phosphorylation analysis, 3D cultures were irradiated with 0 mGy, 50 mGy or 2 Gy and incubated for 1 or 6 hours at 37°C 5% CO_2. Cultures were lysed in ice-cold RIPA buffer (Thermo Scientific, UK) containing protease and phosphatase inhibitors and incubated on ice for 30 min. Cell lysate was passed through a 25G needle at least 10 times to increase nuclear lysis and centrifuged at 14000 g for 20 min at 4°C. The supernatant was collected for analysis. Total protein quantification was performed using Pierce BCA Protein Assay Kit (Thermo Scientific, UK). Immune capillary electrophoresis was performed using Wes Simple^{TM} (ProteinSimple, USA) following manufacturer's instructions.

Mitochondrial redox measurements

A genetically encoded redox-sensitive biosensor fluorescent protein (roGFP2) fused to redox-active protein (Grx1) and with an N-terminal mitochondrial targeting sequence (mito-roGFP2-Grx1), was used for the measurement of glutathione redox potential. Primary mouse keratinocytes (PMOKs) expressing mito-roGFP2-Grx1 were established by retroviral transduction. Briefly, Phoenix-AMPHO cells were transfected with pLPCX/mito-roGFP2-Grx1 using lipofectamine (Life Technologies) following the manufacturer's protocol. 8 hours post- transfection, cells were washed twice with HBSS and fresh medium was added. 48 hours after transfection the supernatant was collected and passed through a 0.44 μm filter. 24 hours before infection PMOKs were seeded at 10^6 cells per insert in a six-well plate (ThinCert^{TM} insert, Greiner Bio-One). PMOKs were incubated with the virus-containing supernatant supplemented with Polybrene (4 μg/ml) for 24 hours at 37°C 5% CO_2. Cells were then grown in medium containing puromycin (0.5 μg/ml) for several days before starting each experiment. PMOKs expressing mito-roGFP2-Grx1 were irradiated with 0 mGy or 50 mGy and incubated for 5, 30 or 60 minutes at 37°C, 5% CO_2. Cells were then treated with 20 mM N-ethylmaleimide (NEM) for 5 min at room temperature prior to

4% PFA fixation for 10 minutes at room temperature. Cells were washed in HBSS twice and incubated with wheat germ agglutinin (WGA) to stain the cell membrane. Confocal 3D images were acquired on a Leica TCS SP8 confocal microscope (× 40 objective; optimal pinhole; speed 400 Hz; line average 1; resolution, 1024 × 1024, optimal step size) using 405nm and 488nm laser lines for excitation and 500-540nm filter for emission collection. 3D images were processed using Imaris (Bitplane) or ImageJ to identify each mitochondria and to calculate 405/488 ratio. Number of mitochondria analyzed for each condition is shown in Table S2. Background subtraction for each channel was performed. For pseudocolor display, the ratio was coded on a spectral color scale ranging from blue (fully reduced) to white (fully oxidized), with limits set by the *in situ* calibration. Calibration was done using 20 mM DTT and 100 μM H_2O_2 treated samples to drive roGFP2 to their fully reduced and fully oxidized forms, respectively.

QUANTIFICATION AND STATISTICAL ANALYSIS

Data are expressed as median values ± SEM unless otherwise indicated. Differences between groups were assessed by 2 tailed unpaired t test or Anova for normally distributed data or 2 tailed Mann-Whitney U test for skewed data, using GraphPad Prism software. For the mitochondrial 405/488 ratios, one-tailed Mann-Whitney U test was used for pairwise comparisons ($\alpha = 0.05$). For paired comparisons of clonal behavior across conditions, Peacock's test was used, a two-dimensional extension of Kolmogorov-Smirnov test (implemented as kstest_2s_2d function for MATLAB) (Peacock, 1983). No statistical method was used to predetermine sample size. The experiments were not randomized. The investigators were not blinded to allocation during experiments and outcome assessment.

Cell Stem Cell, Volume *25*

Supplemental Information

Outcompeting *p53*-Mutant Cells

in the Normal Esophagus

by Redox Manipulation

David Fernandez-Antoran, Gabriel Piedrafita, Kasumi Murai, Swee Hoe Ong, Albert Herms, Christian Frezza, and Philip H. Jones

Supplemental Material for:

Outcompeting p53-mutant cells in normal esophagus by redox manipulation

David Fernandez-Antoran, Gabriel Piedrafita, Kasumi Murai, Swee Hoe Ong, Albert Herms, Christian Frezza, Philip H. Jones

This PDF file includes:

Supplementary Figures and Legends

Figure S1. Related to Figures 2, 5 and 6

Figure S2. Related to Figure 2

Figure S3. Related to Figure 2

Figure S4. Related to Figures 2 and 3

Figure S5. Related to Figure 5

Figure S6. Related to Figure 6

Supplementary Tables and Data

Table S1, related to Figures 3 and 5: Number of quantified mitochondria in Figure 3 and Figure 5

Table S2, related to Figures 2,3,4, 5, 6: Number of quantified clones per condition per time point in Figures 2, 4, 5, 6 and Figure S3.

Supplementary Figures and Legends

Figure S1

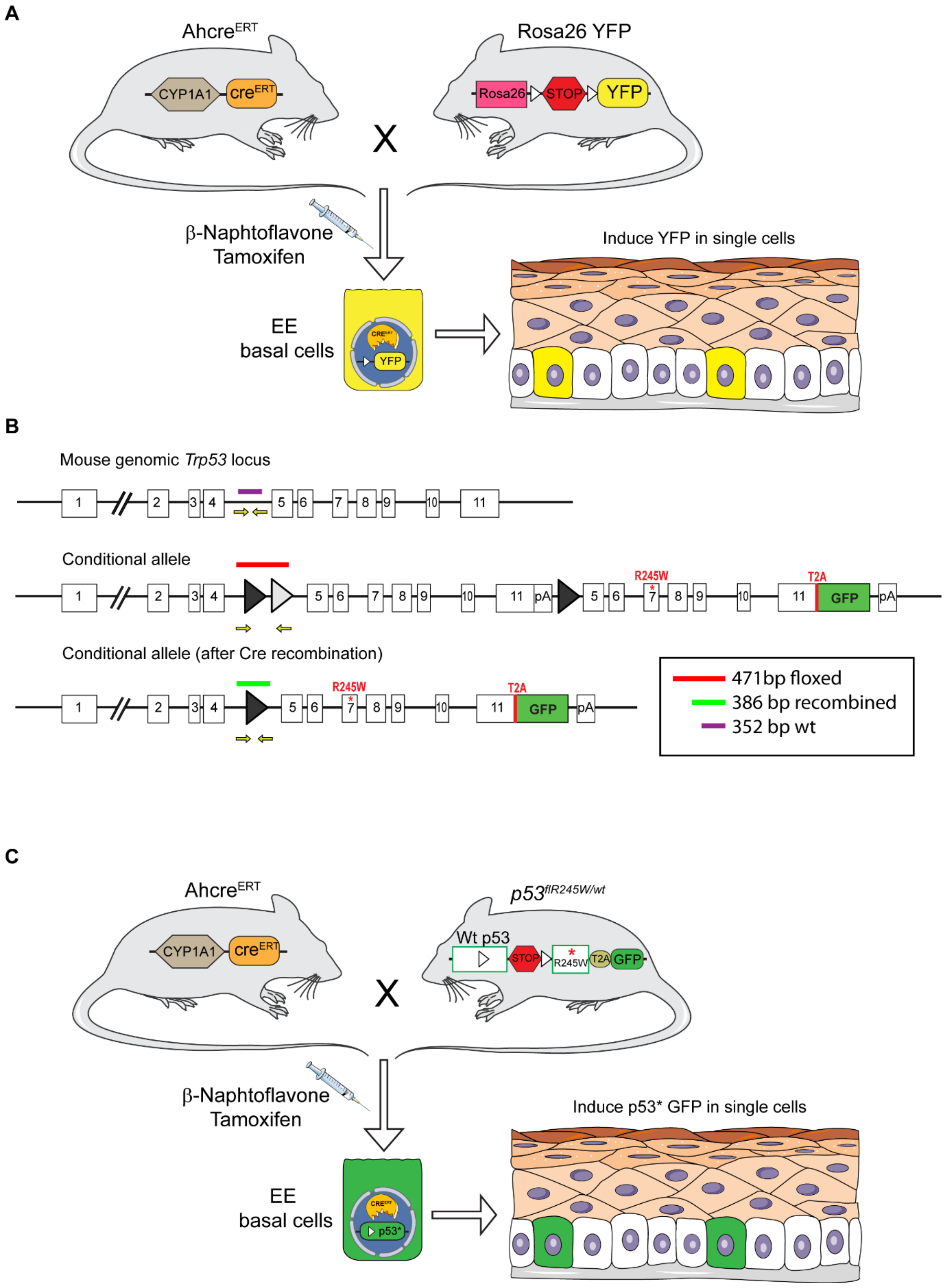

Figure S1. Transgenic mouse models. Related to Figures 2, 5 and 6.

(A) Generation of double transgenic *Ahcre*^{ERT}*R26*$^{flEYFP/wt}$*,* (*RYFP)* mice for lineage cell tracing experiments. *Yfp* (yellow) is targeted to the *Rosa26* locus downstream of a 'stop' cassette (red). Following *cre* induction, the stop cassette is excised and YFP protein expressed in a small proportion of esophageal basal cells. (B) Gene targeting strategy for generation of *p53*$^{R245W\text{-}GFP}$ mice, in which a conditional mutant allele of *Trp53*^{R245W} (*p53*)* linked to the reporter protein GFP is targeted to the *Trp53* locus (Murai et al., 2018). (C) Generation of *Ahcre*^{ERT} *p53** mice. Following *cre* induction, *Trp53* wild type exons 5 to 11 are excised and p53* mutant allele and GFP reporter protein are expressed in a small proportion of oesophageal basal cells.

Figure S2

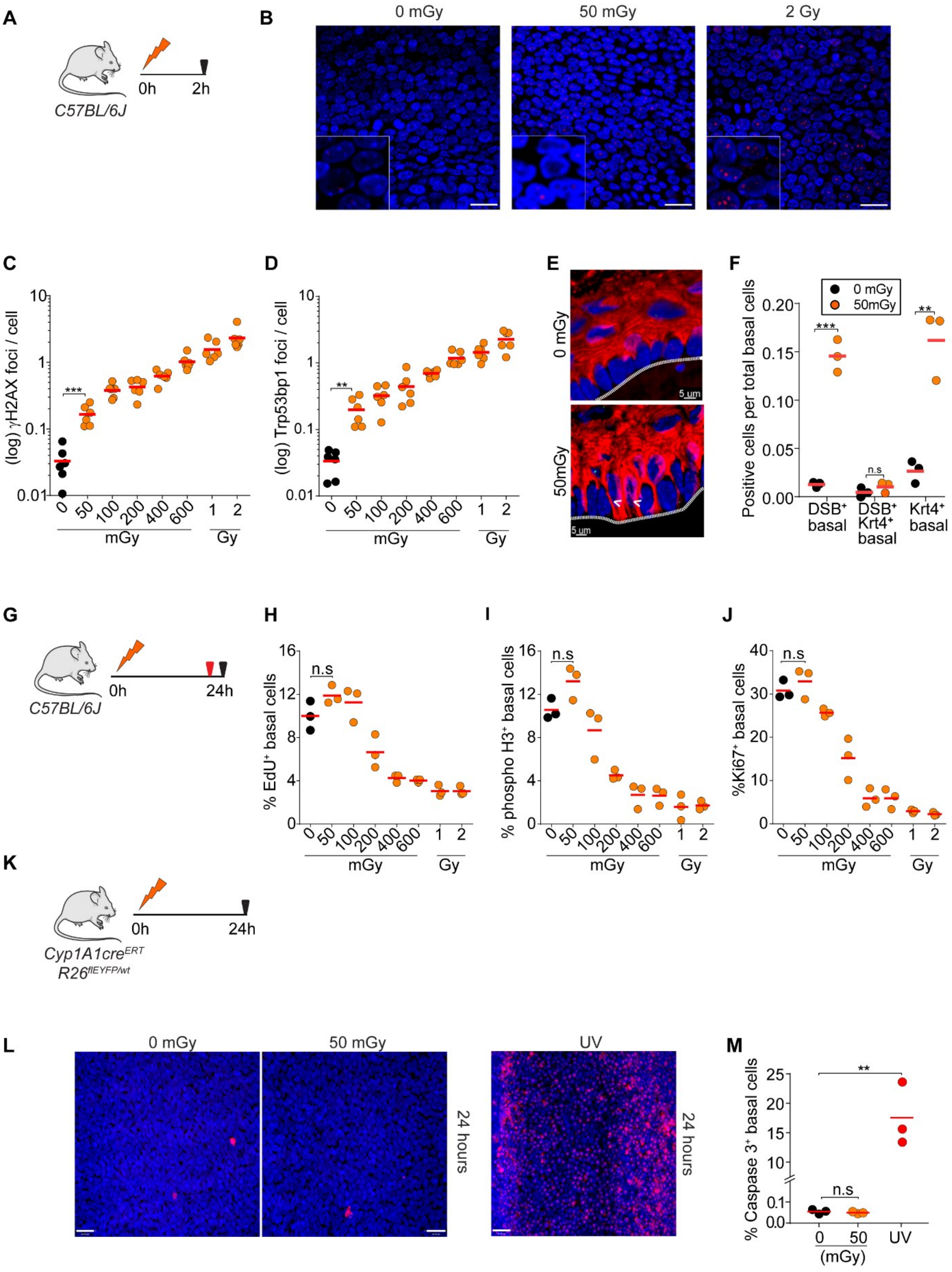
A
C57BL/6J
0h
2h
B
0 mGy
50 mGy
2 Gy
C
(log) γH2AX foci / cell
10
1
0.1
0.01
0
50
100
200
400
600
1
2
mGy
Gy
***
D
(log) Trp53bp1 foci / cell
**
E
0 mGy
50mGy
5 um
F
Positive cells per total basal cells
0 mGy
50mGy
0.20
0.15
0.10
0.05
0.00
***
n.s
**
DSB+ basal
DSB+ Krt4+ basal
Krt4+ basal
G
C57BL/6J
0h
24h
H
% EdU+ basal cells
16
12
8
4
0
n.s
I
% phospho H3+ basal cells
J
%Ki67+ basal cells
40
30
20
10
0
K
Cyp1A1creERT R26flEYFP/wt
0h
24h
L
0 mGy
50 mGy
UV
24 hours
M
% Caspase 3+ basal cells
25
20
15
10
0.1
0.0
**
n.s
0
50
(mGy)
UV

Figure S2. Dose response of EE to whole body ionizing radiation. Related to Figure 2.

(A) Protocol to study DNA damage dose response of basal cells in EE. Wild type *C57BL/6J* mice were irradiated and tissue collected 2 hours later. (B) Top down views of confocal z stacks of *Cyp1A1cre*^{ERT} *Rosa26*$^{flEYFP/wt}$ mice EE wholemounts stained for DNA (DAPI, blue) showing nuclear foci γH2AX (double strand DNA break marker, red) 2 hours after 0 mGy, 50 mGy or 2Gy exposures. DAPI (blue). Scale bars, 20 µm. (C and D) Nuclear foci/basal cell of γH2AX (C) and *Trp53bp1* (D) after whole body irradiation with doses shown. At least 250 basal cells per mouse in 6 mice at each dose were analyzed by confocal imaging. ***p<0.001 and **p<0.01 (unpaired t test). (E) OCT embedded 10 µm thickness cryosections of EE of Cyp1A1^{creERT} Rosa26$^{flEYFP/wt}$ 2 hours after 0 mGy or 50 mGy LDIR. Krt4 (red), Trp53bp1 (DSB, green) and DAPI (blue). Dashed lines indicate basement membrane. Arrowheads show Krt4 positive basal cells. Scale bars, 5µm. (F) Quantification of DSB$^+$, DSB$^+$ Krt4$^+$ and Krt4$^+$ basal cells shown in (E). Points are mean values from individual mice. ***p<0.001, **p<0.01, n.s not significant, (unpaired t test), n=3 mice per condition. (G) Protocol to study the effect of ionizing radiation on cell proliferation. Wild-type *C57BL/6J* mice were irradiated as in (A) and culled 24 hours later. One hour before culling, animals were injected with EdU intraperitoneally. (H-J) Percentage of basal cells positive for the proliferation markers, EdU (H) phospho-Histone 3 (I) and Ki67 (J), 24 hours after exposure to different doses of ionizing radiation. 3 mice per condition were analyzed by confocal imaging. n.s not significant (unpaired t test). (K-M) Apoptosis *in vivo* assay after LDIR. (K) Experimental protocol. *Cyp1A1cre*^{ERT}*Rosa26*$^{flEYFP/wt}$ mice were irradiated with 0 or 50 mGy LDIR and EE stained 24 hours after LDIR exposure. As a positive control tissue was exposed to ultraviolet radiation and maintained in explant culture for 24 hours prior to staining (Clayton et al., 2007). (L) Confocal images of basal layer of EE stained for activated Caspase 3 (red) and DAPI (blue). Scale bars, 30 µm. (M) Quantification of the percentage of Caspase 3$^+$ cells shown in (J). At least 3000 basal cells in 3 mice were analyzed per condition by confocal imaging. ** p<0.01, n.s. not significant (unpaired t test).

Figure S3

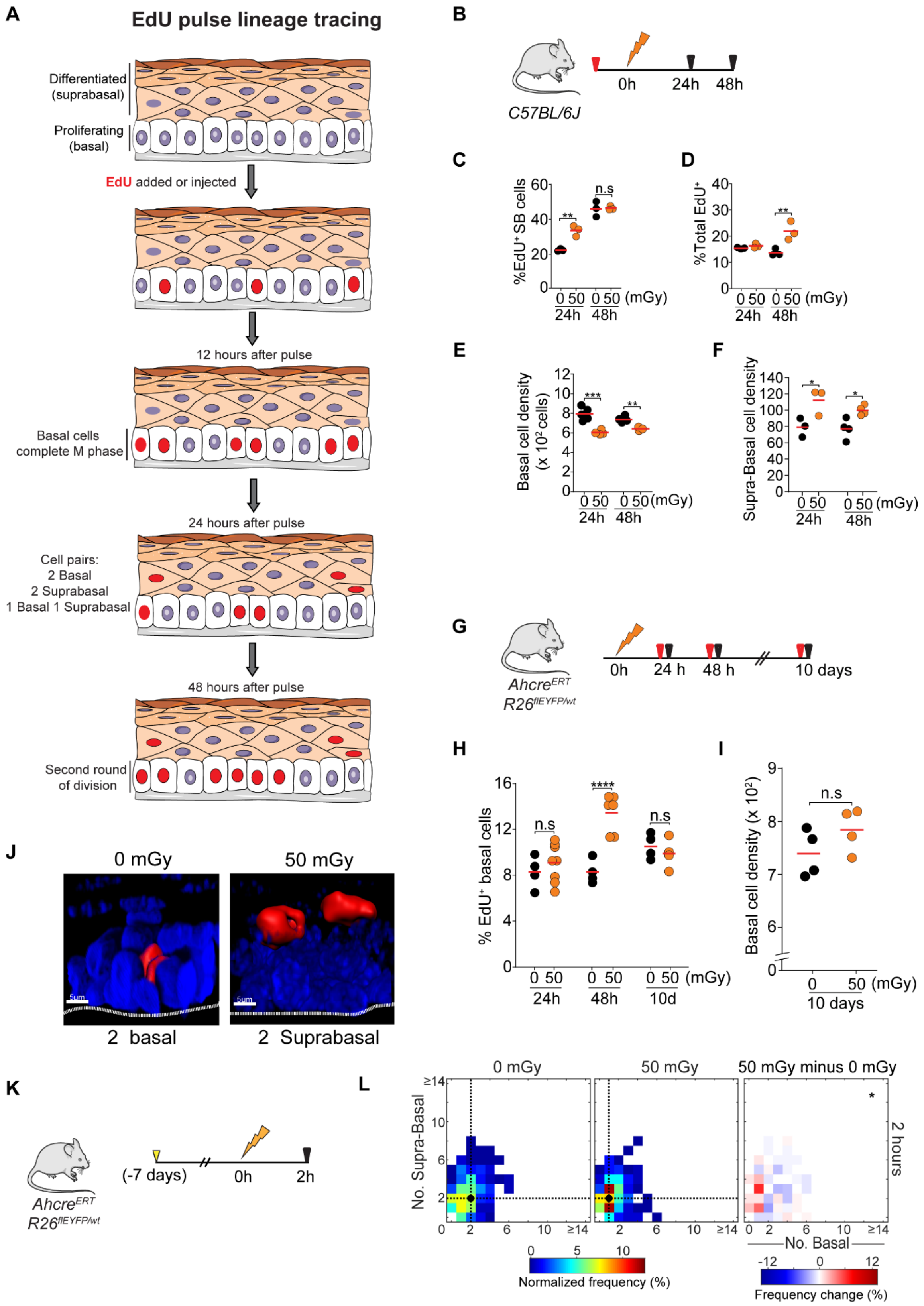

A
EdU pulse lineage tracing
Differentiated (suprabasal)
Proliferating (basal)
EdU added or injected
12 hours after pulse
Basal cells complete M phase
24 hours after pulse
Cell pairs:
2 Basal
2 Suprabasal
1 Basal 1 Suprabasal
48 hours after pulse
Second round of division
B
C57BL/6J
0h
24h
48h
C
%EdU+ SB cells
n.s
(mGy)
D
%Total EdU+
E
Basal cell density (x 10² cells)
F
Supra-Basal cell density
G
AhcreERT R26flEYFP/wt
0h
24 h
48 h
10 days
H
% EdU+ basal cells
24h
48h
10d
I
Basal cell density (x 10²)
10 days
J
0 mGy
50 mGy
2 basal
2 Suprabasal
K
(-7 days)
0h
2h
L
0 mGy
50 mGy
50 mGy minus 0 mGy
No. Supra-Basal
No. Basal
2 hours
Normalized frequency (%)
Frequency change (%)

Figure S3. EdU lineage tracing following low dose radiation. Related to Figure 2.

(A) EdU pulse lineage cell tracing. Cartoon shows where EE EdU labelled cells (red) are located at different time points after EdU labelling. (B) Experimental protocol. Mice were given EdU 1 hour before irradiation (red triangle) to allow number and location of EdU^+ cells in EE to be tracked by imaging 24 and 48 hours post irradiation. (C-F) Percentage of EdU^+ suprabasal (C) EdU^+ total cells (D) basal cell density (E) and supra-basal cell density (F) 24 and 48 hours after 0 or 50 mGy LDIR. Points are mean values from individual mice. n=3, 4 or more mice per condition **** p<0.0001, *** p<0.001, **p<0.01, *p<0.05, n.s. not significant (unpaired t test). Total cells per condition in (C) was >4000, >6000 in (D) and (E) and >3500 basal cells in (F). (G) Experimental protocol. *Cyp1A1cre*^{ERT}*Rosa26*$^{flEYFP/wt}$ mice were irradiated with 0 or 50 mGy LDIR (black arrows) and were given EdU (red arrows) 1 hour before culling for each time point. EdU^+ basal cells were detected by confocal imaging. (H) Percentage of EdU^+ basal cells at time points shown in (G) ****p<0.0001, n.s. not significant, (unpaired t test). (I) Basal cell density (as number of cells per field of view) 10 days after 0 or 50 mGy LDIR. At least 2900 basal cells were quantified per condition in 4 mice per condition. (J) Rendered confocal z stacks showing side views of typical EdU^+ clones found after LDIR, containing two basal cells (for 0 mGy) and two suprabasal cells (for 50 mGy). EdU^+ (red), DAPI (blue). Dashed lines indicate basement membrane, scale bars, 5 µm. (K) Experimental protocol: *cre* was induced 7 days before irradiation in *Cyp1A1cre*^{ERT}*Rosa26*$^{flEYFP/wt}$ reporter mice (RYFP), yellow arrow. Samples were collected at 2 hours post irradiation and clones size were analyzed. (L) Heat maps representing frequency of clones with the number of basal and supra-basal cells indicated and differences between 0 and 50 mGy irradiated animals (right hand panel) at 2 hours. Black dots and dashed lines indicate geometric median clone-size. *p = 0.042 (Peacock's test). n = 300 clones per condition.

Figure S4

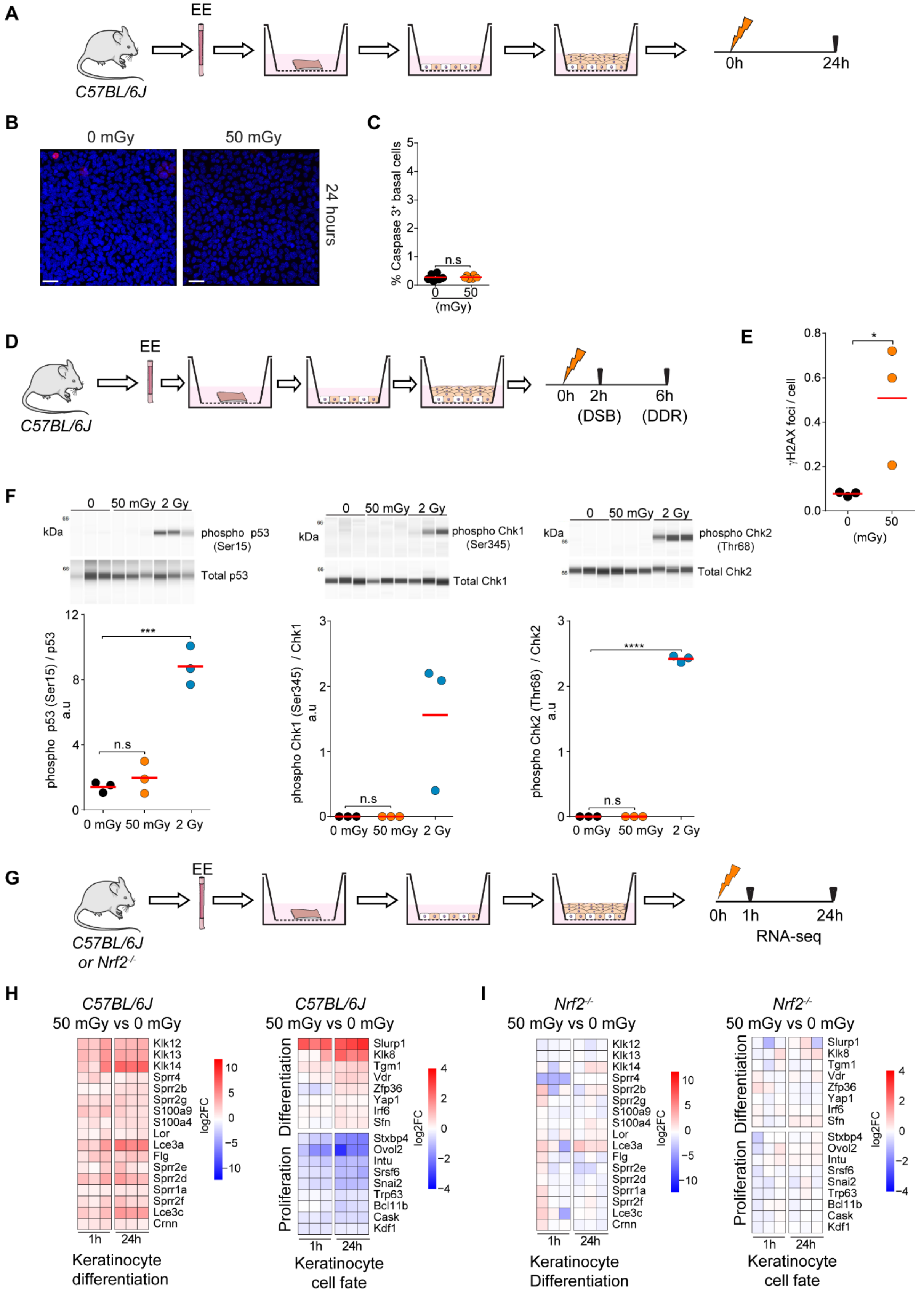

A
EE
C57BL/6J
0h
24h
B
0 mGy
50 mGy
24 hours
C
% Caspase 3+ basal cells
n.s
(mGy)
D
C57BL/6J
0h 2h 6h
(DSB) (DDR)
E
γH2AX foci / cell
(mGy)
F
0 50 mGy 2 Gy
kDa
phospho p53 (Ser15)
Total p53
phospho Chk1 (Ser345)
Total Chk1
phospho Chk2 (Thr68)
Total Chk2
phospho p53 (Ser15) / p53 a.u
phospho Chk1 (Ser345) / Chk1 a.u
phospho Chk2 (Thr68) / Chk2 a.u
0 mGy 50 mGy 2 Gy
G
C57BL/6J or Nrf2-/-
0h 1h 24h
RNA-seq
H
C57BL/6J
50 mGy vs 0 mGy
Klk12 Klk13 Klk14 Sprr4 Sprr2b Sprr2g S100a9 S100a4 Lor Lce3a Flg Sprr2e Sprr2d Sprr1a Sprr2f Lce3c Crnn
log2FC
1h 24h
Keratinocyte differentiation
Slurp1 Klk8 Tgm1 Vdr Zfp36 Yap1 Irf6 Sfn
Stxbp4 Ovol2 Intu Srsf6 Snai2 Trp63 Bcl11b Cask Kdf1
Differentiation
Proliferation
Keratinocyte cell fate
I
Nrf2-/-
50 mGy vs 0 mGy
Keratinocyte Differentiation
Keratinocyte cell fate

Figure S4. Effects of 50mGy irradiation on primary esophageal 3D cultures. Related to Figures 2 and 3.

(A) Experimental protocol. Primary mouse keratinocyte 3D cultures were irradiated with 0 or 50 mGy LDIR and analyzed for Caspase3^{+} basal cells 24 hours later. (B) Top down views of confocal z stacks of typical *in vitro* primary keratinocyte 3D cultures from *C57BL/6J* mice stained for Caspase 3^{+} (red). DAPI (blue). Scale bars, 30 µm. (C) Percentage of Caspase 3^{+} basal cells in (B). n.s not significant (unpaired t test). n=6 mice. (D) Experimental protocol. Cultures were irradiated with 0 or 50 mGy LDIR or 2Gy and stained with the double strand DNA break marker γH2AX, 2 hours after irradiation. Staining visualized by confocal microscopy. (E) Number of γH2AX foci per cell in primary keratinocyte 3D cultures from (D) * p<0.05 (unpaired t-test). n= 3 mice per condition. At least 4000 basal cells per condition were quantified. (F) Quantitative immuno-capillary electrophoresis analysis of DNA damage response (DDR) pathway key phosphorylation events (phospho p53 Ser15, phospho CHK1 Ser345 and phospho CHK2 Thr68) for primary keratinocyte 3D cultures shown in (D) 6 hours after irradiation. 2 Gy irradiated samples were included as positive control of DDR pathway phosphorylation. n.s. not significant, **** p<0.0001, *** p<0.001 (unpaired t-test). n= 3 mice per condition. (G) Experimental protocol. 3D cultures from *C57BL/6J* and *Nrf2*$^{-/-}$ animals were irradiated with 0 or 50 mGy LDIR and total RNA was isolated from samples 1 hour and 24 hours after irradiation. (H and I) RNA-seq generated heat maps, performed on biological triplicate cultures, 1 and 24 hours after 50 mGy LDIR compared to 0 mGy, for *C57BL/6J* (H) and *Nrf2*$^{-/-}$ (I) focusing on transcripts with adjusted p<0.05, involved in keratinocyte differentiation (first panel), and keratinocyte cell fate regulation (second panel).

Figure S5

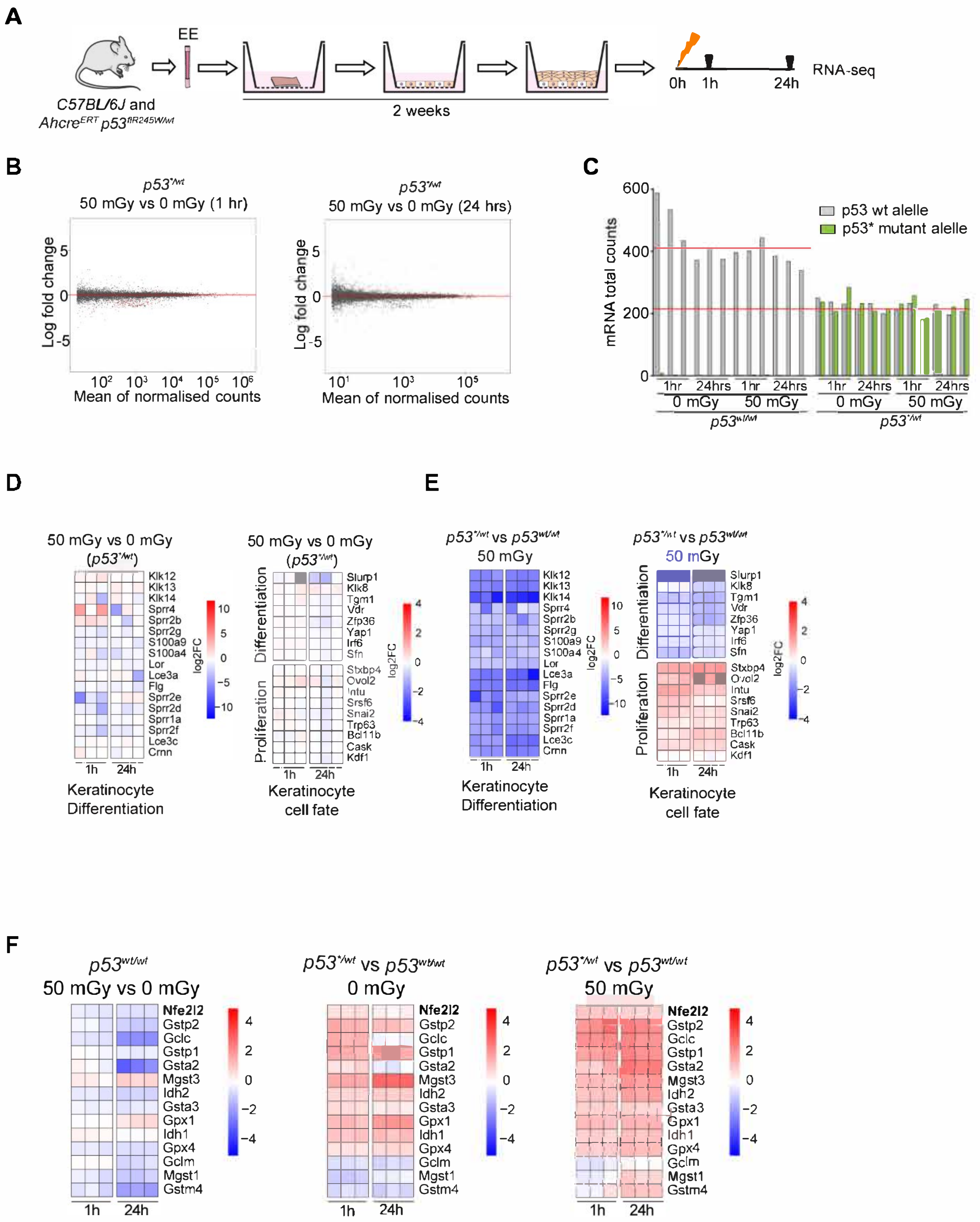
A
EE
0h
1h
24h
RNA-seq
C57BL/6J and
AhcreERT p53flR245W/wt
2 weeks
B
p53*/wt
50 mGy vs 0 mGy (1 hr)
50 mGy vs 0 mGy (24 hrs)
Log fold change
Mean of normalised counts
C
mRNA total counts
p53 wt allele
p53* mutant allele
1hr
24hrs
0 mGy
50 mGy
p53wt/wt
p53*/wt
D
50 mGy vs 0 mGy
(p53*/wt)
Klk12
Klk13
Klk14
Sprr4
Sprr2b
Sprr2g
S100a9
S100a4
Lor
Lce3a
Flg
Sprr2e
Sprr2d
Sprr1a
Sprr2f
Lce3c
Crnn
log2FC
1h
24h
Keratinocyte
Differentiation
Differentiation
Proliferation
Slurp1
Klk8
Tgm1
Vdr
Zfp36
Yap1
Irf6
Sfn
Stxbp4
Ovol2
Intu
Srsf6
Snai2
Trp63
Bcl11b
Cask
Kdf1
Keratinocyte
cell fate
E
p53*/wt vs p53wt/wt
50 mGy
F
p53wt/wt
50 mGy vs 0 mGy
p53*/wt vs p53wt/wt
0 mGy
50 mGy
Nfe2l2
Gstp2
Gclc
Gstp1
Gsta2
Mgst3
Idh2
Gsta3
Gpx1
Idh1
Gpx4
Gclm
Mgst1
Gstm4

Figure S5. Transcriptional profile of *p53*$^{*/wt}$ primary esophageal 3D cultures after 50mGy LDIR. Related to Figure 5.

(A) Protocol. Primary keratinocyte 3D cultures from *C57BL/6J* (*p53*$^{wt/wt}$) and *p53*$^{R245W\text{-}GFP/wt}$ (*p53*$^{*/wt}$) mice were irradiated with 0 or 50 mGy LDIR and total RNA was isolated from samples 1 hour and 24 hours after irradiation. (B) MA plots of RNA-seq data of cultures from *p53*$^{*/wt}$ mice, comparing irradiated and unirradiated cultures at times shown, red indicates transcripts with adjusted $p<0.05$. (C) mRNA total counts (expression levels) from RNA-seq data for *p53* alleles in induced wild-type or *p53*$^{*/wt}$ cultures. Bars indicate counts for *p53* wild type (grey) and *p53** (green) mRNAs, red lines show mean for each allele. (D and E) Heat maps from RNA-seq performed on *p53*$^{*/wt}$ biological triplicate cultures, 1 and 24 hours after 50 mGy LDIR compared with 0 mGy (D), and *p53*$^{*/wt}$ versus *p53*$^{wt/wt}$ after 50 mGy LDIR (E) focusing on transcripts with adjusted $p<0.05$, involved in keratinocyte differentiation and keratinocyte cell fate regulation. (F) RNA-seq generated heat maps, performed on *p53*$^{wt/wt}$ and *p53*$^{wt/*}$ 3D cultures showing transcript expression differences with adjusted $p<0.05$ for the indicated comparisons, directly regulated by *Nrf2* (*Nfe2l2*).

Figure S6

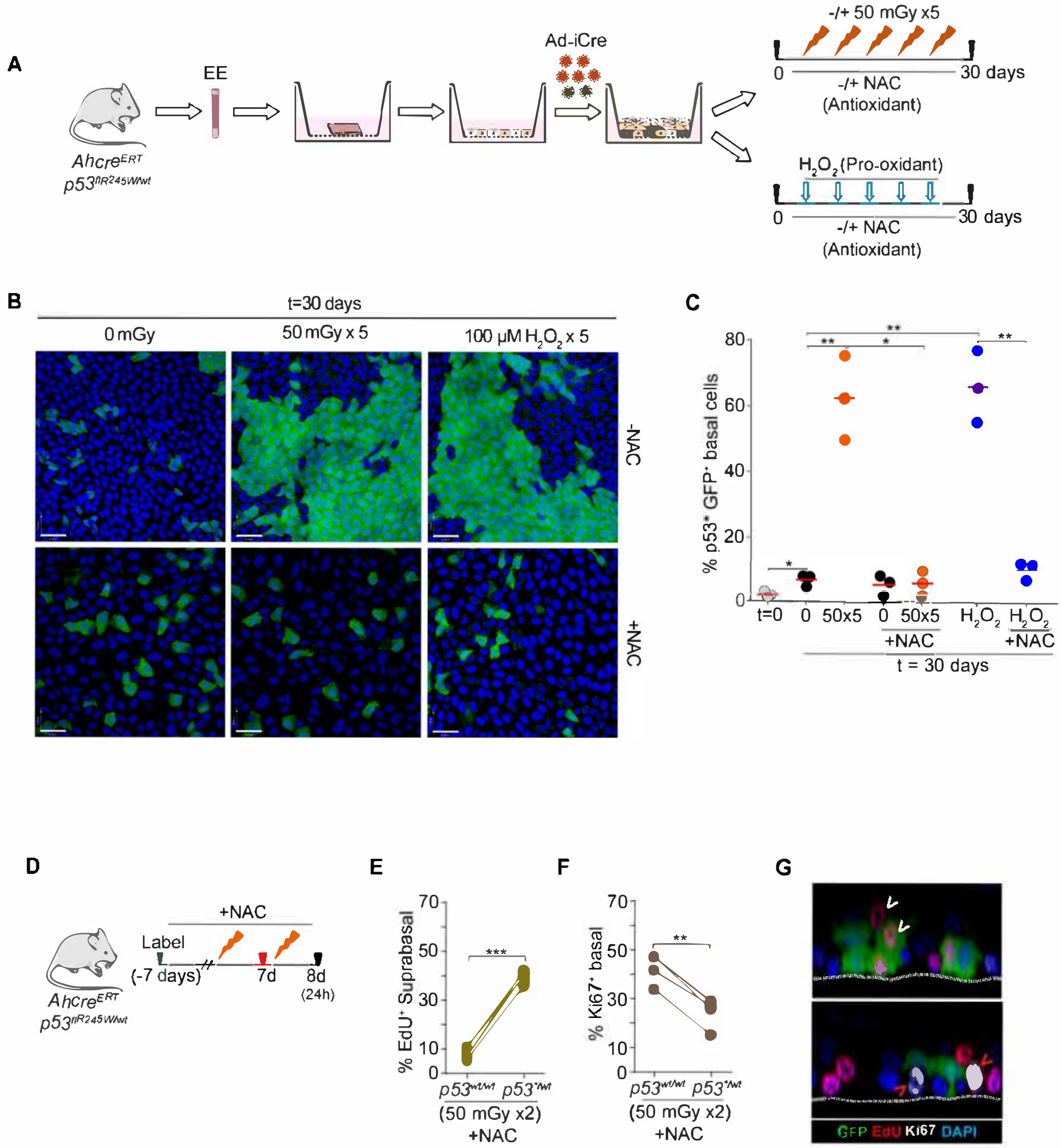

A
AhcreERT
p53flR245W/wt
EE
Ad-iCre
-/+ 50 mGy x5
0
30 days
-/+ NAC
(Antioxidant)
H2O2 (Pro-oxidant)
0
30 days
-/+ NAC
(Antioxidant)
B
t=30 days
0 mGy
50 mGy x 5
100 µM H2O2 x 5
-NAC
+NAC
C
% p53* GFP+ basal cells
80
60
40
20
0
t=0
0
50x5
0
50x5
+NAC
H2O2
H2O2
+NAC
t = 30 days
D
+NAC
Label
(-7 days)
7d
8d
(24h)
AhcreERT
p53flR245W/wt
E
% EdU+ Suprabasal
70
50
30
10
0
***
p53wt/wt
p53*/wt
(50 mGy x2)
+NAC
F
% Ki67+ basal
70
50
30
10
0
**
p53wt/wt
p53*/wt
(50 mGy x2)
+NAC
G
GFP EdU Ki67 DAPI

Figure S6. Effects of 50mGy LDIR and antioxidant treatment on p53*/wt and p53wt/wt in vitro and in vivo. Related to Figure 6.

(A) Experimental protocol. Primary keratinocyte 3D cultures from *p53*$^{R245W-GFP/wt}$ mice were induced at clonal level by adenoviral cre infection and exposed to five doses of 50 mGy LDIR or five doses of 100µM H_2O_2 with or without NAC treatment. (B) Top down views of confocal z stacks of primary 3D cultures described in (A) showing p53$^{*/wt}$ clones (green), 30 days after different exposures and treatments. Basal layer cells are shown. DAPI (blue). Scale bars, 36 µm.

(C) Percentage of *p53*$^{*/wt}$ basal cells shown in (B). ** $p<0.01$, * $p<0.05$, n.s $p>0.05$ (unpaired t-test). At least 2900 basal cells were quantified per condition. n=3 (independent cultures from different mice). (D) *p53*$^{R245W-GFP/wt}$ mice (*p53*/wt*) were induced to give expression of p53* in single cells. 7 days later, animals were irradiated with a course of 2 doses of 50 mGy LDIR over 2 weeks. (E and F) Percentage of EdU+ suprabasal cells, (E) and Ki67+ basal cells (F) 24 and 48 hours after 2 doses of 50 mGy in the presence of NAC, in *p53*$^{wt/wt}$ and *p53*$^{*/wt}$ areas within the same animal. Lines link same mouse. ***$p<0.001$, ** $p<0.01$ (paired t-test). n= 4 mice per time point. At least 28000 basal cells were analyzed for each time point.

(G) Representative sections of EE whole mounts from p53$^{*/wt}$ mice, 48 hours after 50 mGy LDIR in the presence of NAC, showing EdU+ suprabasal cells (red, marked with white arrows) in *p53** clones and Ki67+ basal cells (white, marked with red arrows) in adjacent *p53*$^{wt/wt}$ cells.

Supplementary Tables and Data

Table S1: Related to Figures 3 and Figure S5.

Fig. 3
Number of quantified mitochondria
(see also data shown in Table S4).

WT	0 mGy	50 mGy
5 min	238521	114768
30 min	197460	105705
60 min	52204	55347
DTT	47347	
H2O2	75413	

WT	0 mGy	0 mGy +DTT	50 mGy	50 mGy+DTT
5 min	63477	50067	66319	66253
30 min	48204	56192	64836	68113
60 min	68880	43877	45182	62252

Fig.S5
Number of quantified mitochondria
(see also data shown in Table S3).

	0 mGy		50 mGy	
	$p53^{wt/wt}$	$p53^{*/wt}$	$p53^{wt/wt}$	$p53^{*/wt}$
5 min	238521	43491	114768	35301
30 min	197450	103688	105705	14730
60 min	52204	43243	55347	7171
DTT	47347	30346		
H2O2	75413	18598		

Table S2: Related to Figures 2, 4-6 and Figure S3.

Number of quantified clones per condition per time point

N= Number of mice per condition per time point

Figure 2D		
	0 mGy	50 mGy
24h	1000	1000
48h	1530	1800

N=8

Figure 4E		
	0 mGy	50 mGy
24h	600	600
48h	600	600

N=4

Figure 5C		
	0 mGy	50 mGy x1
48h	150	450

N=4

Figure 5H and 6E		
	0 mGy	50 mGy x5
-NAC	300	300
+NAC	300	300

N=3

Figure S3L		
	0 mGy	50 mGy x1
2 hours	300	300

N=3
